# Phenotypes in Brugada syndrome with different genotypes triggered by fever or inflammation using gene-edited iPSCs

**DOI:** 10.1186/s13287-025-04793-6

**Published:** 2025-12-01

**Authors:** Yingrui Li, Lena Rose, Timo Prädel, Mandy Kleinsorge, Xuehui Fan, Zenghui Meng, Chen Yan, Rui Liu, Xinhao Lei, Binyi Zhao, Guoqiang Yang, Zhenxing Liao, Hendrik Dinkel, Alexandra Viktoria Busley, Rujia Zhong, Feng Zhang, Qiang Xu, Lasse Maywald, Assem Aweimer, Mengying Huang, Alexander Moscu-Gregor, Nazha Hamdani, Luca Schneider, Yeweynwuha Zemedi, Saltanat Zhazykbayeva, Alyssa Hohn, Zhen Yang, Lin Qiao, Andreas Mügge, Lukas Cyganek, Xiaobo Zhou, Ibrahim Akin, Ibrahim El-Battrawy

**Affiliations:** 1 First Department of Medicine, Faculty of Medicine Mannheim, University Medical Centre Mannheim (UMM), Heidelberg University, Theodor-Kutzer-Ufer 1-3, 68167 Mannheim, Germany; 2 Key Laboratory of Medical Electrophysiology of Ministry of Education and Medical Electrophysiological Key Laboratory of Sichuan Province, Institute of Cardiovascular Research, Southwest Medical University, Luzhou, 646000 Sichuan China; 3 Department of Cardiology, The Second Affiliated Hospital of Chongqing Medical University, Chongqing, 400010 China; 4 Stem Cell Unit, Clinic for Cardiology and Pneumology, University Medical Center Göttingen, 37075 Göttingen, Germany; 5 DZHK (German Center for Cardiovascular Research), Partner Site Heidelberg-Mannheim, 68167 Mannheim, Germany; 6 DZHK (German Center for Cardiovascular Research), Partner Site Göttingen, 37075 Göttingen, Germany; 7 Department of Cardiology and Angiology, Bergmannsheil University Hospitals, Ruhr University of Bochum, 44789 Bochum, Germany; 8 Center for Human Genetics and Laboratory Medicine, Martinsried, Germany; 9 Department of Cellular and Translational Physiology, Institute of Physiology, Ruhr University, Bochum, Germany; 10 Institut für Forschung und Lehre (IFL), Molecular and Experimental Cardiology, Ruhr University, Bochum, Germany

**Keywords:** Brugada syndrome, Induced pluripotent stem cells, Hyperthermia, Inflammation

## Abstract

**Background:**

Fever or inflammation state may enhance the Brugada syndrome (BrS) phenotype in some but not all patients. However, the underlying mechanism in human cardiomyocytes has not yet been clarified.

**Methods:**

Human induced pluripotent stem cell (hiPSC) lines generated from fibroblasts of three BrS patients harboring variants in *SCN10A* (abbreviated as BrS1) and *CACNB2* (abbreviated as BrS2), *SCN5A* (abbreviated as BrS3) and one healthy donor (abbreviated as WT) and a site-corrected (using CRISPR/Cas9) hiPSC line of each BrS patient (abbreviated as isogenic1, isogenic2 and isogenic3) were used for differentiation into cardiomyocytes (hiPSC-CMs). Western blot, patch clamp and calcium transient analyses were carried out.

**Results:**

All 3 BrS cell lines showed a significantly reduced peak sodium current (I_Na_) compared with isogenic or WT cells at baseline. Hyperthermia challenge (40 °C) significantly decreased I_Na_ and enhanced arrhythmogeneity in BrS1 and BrS3 but not in BrS2 cells. The hyperthermia effects involved PKA reduction. The lipopolysaccharide (LPS) challenge exacerbated the phenotype in electrophysiological characteristics in all 3 BrS cell lines. ROS-Blocker abolished the LPS effects in all BrS hiPSC-CMs, while an interleukin-6 receptor blocker abolished the proarrhythmic effect of LPS in BrS1 and BrS3 hiPSC-CMs but not in hiPSC-CMs of BrS2.

**Conclusions:**

Hyperthermia exacerbated the BrS phenotype in hiPSC-CMs carrying SCN10A and SCN5A variants, whereas LPS aggravated the phenotype in all three BrS variants through distinct mechanisms; Hyperthermia and LPS effects on BrS phenotype may be genotype-dependent.

**Graphical abstract:**

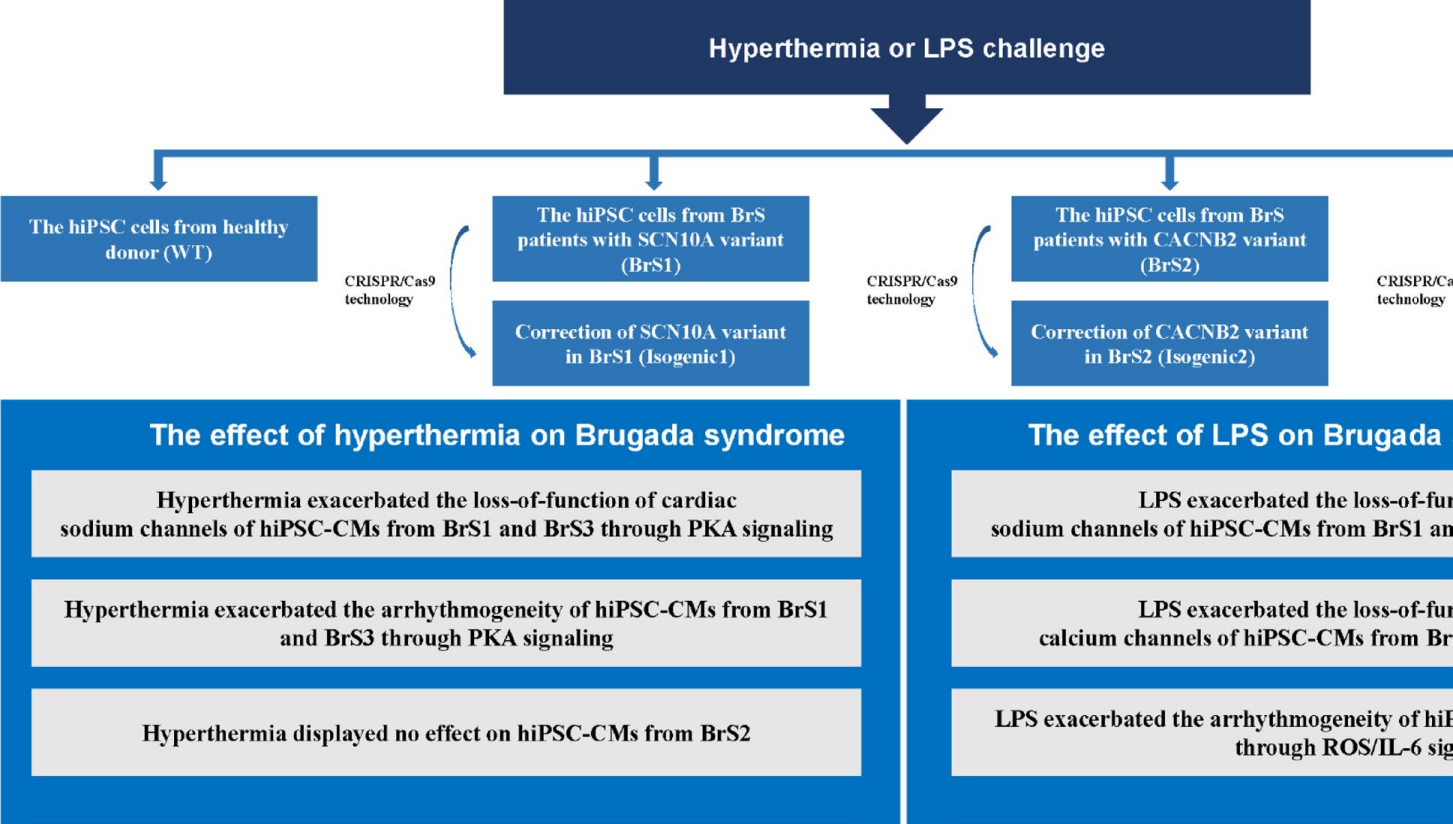

**Supplementary Information:**

The online version contains supplementary material available at 10.1186/s13287-025-04793-6.

## Introduction

Brugada syndrome (BrS) is an inherited arrhythmic disease which is characterized by an elevation of ST-segment in the right precordial leads V1 to V3 of electrocardiogram (ECG). BrS patients may manifest ventricular fibrillation (VF) at any age, usually during rest or sleep [1]. Fever can unmask the typical ECG pattern in patients with BrS, and may increase the risk for malignant arrhythmias [1, 2].

Although the pathological mechanism of BrS is still disputed, cardiac electrophysiological abnormalities in patients with BrS are not generally controversial, especially the loss of function of sodium channels. To date, around 11–28% of BrS patients were found to be carriers of genetic variants, and several genes were reported to be associated with BrS [3]. Most variants are located in the *SCN5A* gene, which encodes the α-subunit of the cardiac sodium channel Nav1.5. Other genes, including *SCN10A* and *CACNB2*, which encodes the sodium channels Nav 1.8 and the β subunit of voltage-gated calcium channels Cav1.2 in the heart respectively, were responsible for 2% to 5% of BrS cases [4], although these genes are still disputed regarding their impact on the BrS phenotype.

Inflammation is commonly the culprit of fever and plays an important role in arrhythmic events, such as malignant ventricular arrhythmias [5, 6]. A case report study displayed that fever caused by SARS-CoV-2 infection could trigger a coved ST-segment elevation in V1-V2 [7]. Another cellular electrophysiological study also showed that febrile illness could further augment arrhythmias in cells with *SCN5A* variant [8]. In addition, several studies also showed the possible relationship between inflammation and BrS [9, 10]. Additionally, as one of the inflammatory factors, interleukin-6 (IL-6) was increased in severely affected coronavirus disease-2019 (COVID-19) patients [11, 12], and could significantly increase the risk of arrhythmic events via affecting cardiac ion channel functions [13, 14]. A recent study displayed that IL-6 induced severe bradycardia, conduction abnormalities, QTc prolongation and asystole in guinea pigs [15]. The inhibitor of the IL-6 receptor tocilizumab (TCZ) could attenuate these electrocardiographic abnormalities [15], suggesting one possible pathomechanism underlying the development of the BrS phenotype and the increase in the risk of arrhythmias during infection.

While several studies have pointed to the involvement of inflammation and fever in BrS, the exact mechanisms remain poorly understood. Besides, whether the impacts of inflammation/fever on BrS is genotype-dependent is so far not clarified. Our recent studies detected fever-related changes in BrS phenotype and effects of lipopolysaccharide (LPS) on peak I_Na_ in hiPSC-CMs from a BrS-patient with an SCN5A variant [16, 17]. However, it remains unclear whether the effects of fever and inflammation differ in BrS patients with different genetic variants. This gap in knowledge necessitates further investigation to understand how BrS with different genotypes respond to fever and inflammatory stimuli at the molecular level.

This study aims to investigate and compare the effects of hyperthermia and inflammation on BrS, focusing on hiPSC-CMs from BrS patients carrying distinct genetic variants (*SCN10A* or *SCN5A* or *CACNB2*). For understanding the differential impact of these stimuli on BrS with various mutations, we aim to uncover the underlying mechanisms contributing to arrhythmogenesis in BrS, which may inform future therapeutic strategies for patients with these genetic mutations.

## Methods

### Ethics

Primary somatic cells were derived from skin biopsies or blood samples after written informed consent had been obtained. Patients regularly underwent a broad diagnostic including echocardiography, ECG, ajmaline test, stress ergometry, and in cases with a suspected myocarditis cardiac magnetic resonance imaging. The study was approved by the Ethik-Kommission II der Universität Heidelberg, Medizinische Fakultät Mannheim (Investigation of the pathophysiology of cardiac arrhythmias using cardiomyocytes, vascular lining cells, nerve cells, and muscle cells from human induced pluripotent stem cells (hiPSCs) in various cardiovascular diseases, 2024 − 573, July 9, 2024). The study was conducted based on the Declaration of Helsinki 1975 of the World Medical Association, as revised in 2013.

A detailed description of methods used for the study is provided in the online supplementary information. Wild type hiPSC line UMGi130-A clone 5 (isWT11.5, here abbreviated as WT) was generated from peripheral mononuclear blood cells of a healthy male donor using the integration-free Sendai virus and described previously [18]. The hiPSC cell lines UMGi126-A clone 23 (isBrSd1.23, here abbreviated as BrS1), UMGi119-A clone 1 (isBrSb2.1, here abbreviated as BrS2) and UMGi127-A clone 1 (isBrSe1.1, here abbreviated as BrS3) were generated from skin fibroblasts of BrS patients with *SCN10A* variant (NM_006514.4: c.3803G >A/p.R1268Q), *CACNB2* variant (NM_000724.4: c.425 C >T/p.S142F), and *SCN5A* variant (NM_000335.5: c.3148G >A/p.A1050T) respectively, using the integration-free Sendai virus and described previously [17, 19, 20]. The variants in *SCN10A*, *CACNB2* and *SCN5A* were corrected by using CRISPR/Cas9 technology. CRISPR-corrected isogenic iPSC lines UMGi126-A-1 clone 53 (isBrSd1-corr.53, here abbreviated as isogenic 1), UMGi119-A-1 clone 6 (isBrSb2-corr.6, here abbreviated as isogenic 2) and UMGi127-A-1 clone 2G6 (isBrSe1-corr.2G6, here abbreviated as isogenic 3) underwent pluripotency characterization, as previously described [17, 21]. The hiPSC lines were differentiated into ventricular cardiomyocytes via WNT signaling modulation and subsequent metabolic selection and cultured for at least 50 days, as previously described [22]. Only cardiomyocytes that were at least at day 50 to 60 of differentiation were used for the experiments in this study to ensure adequate gene expression and protein biosynthesis of cardiac genes. Western blot, patch clamp, calcium imaging and enzyme-linked immunosorbent assay were performed for the study. The details of patch clamp analysis were described in our recent studies [19, 23].

### Statistical analysis

All data, if not otherwise stated, are shown as mean ± SEM and were analyzed by SigmaPlot 14.0 (Systat GmbH, Germany). By analyzing the data with the Normality test, it was decided whether parametric or non-parametric tests were used for analysis. Unpaired student’s t-test analysis was conducted to compare two independent groups with normal distribution. One-way analysis of variance (ANOVA) followed with Holm-Sidak post-test for multiple comparisons was used for comparing more than two groups. The Fisher-test was applied for comparing categorical variables. p values < 0.05 were considered statistically significant.

## Results

### Changes of action potentials and I_Na_ of BrS cell lines

The hiPSC-CMs were generated from 1 healthy donor and 3 BrS patients with *SCN10A*, *CACNB2*, and *SCN5A* variants, respectively. Moreover, the site-corrected isogenic cell lines of each variant were generated by using CRISPR/Cas9 genome editing (figure S2). The hiPSCs expressed the pluripotency markers and showed human embryonic stem cell morphology (figure S2). The hiPSC-CMs displayed cardiac-specific markers. These results were showed in our recent studies [19, 20].

**Fig. 1:**
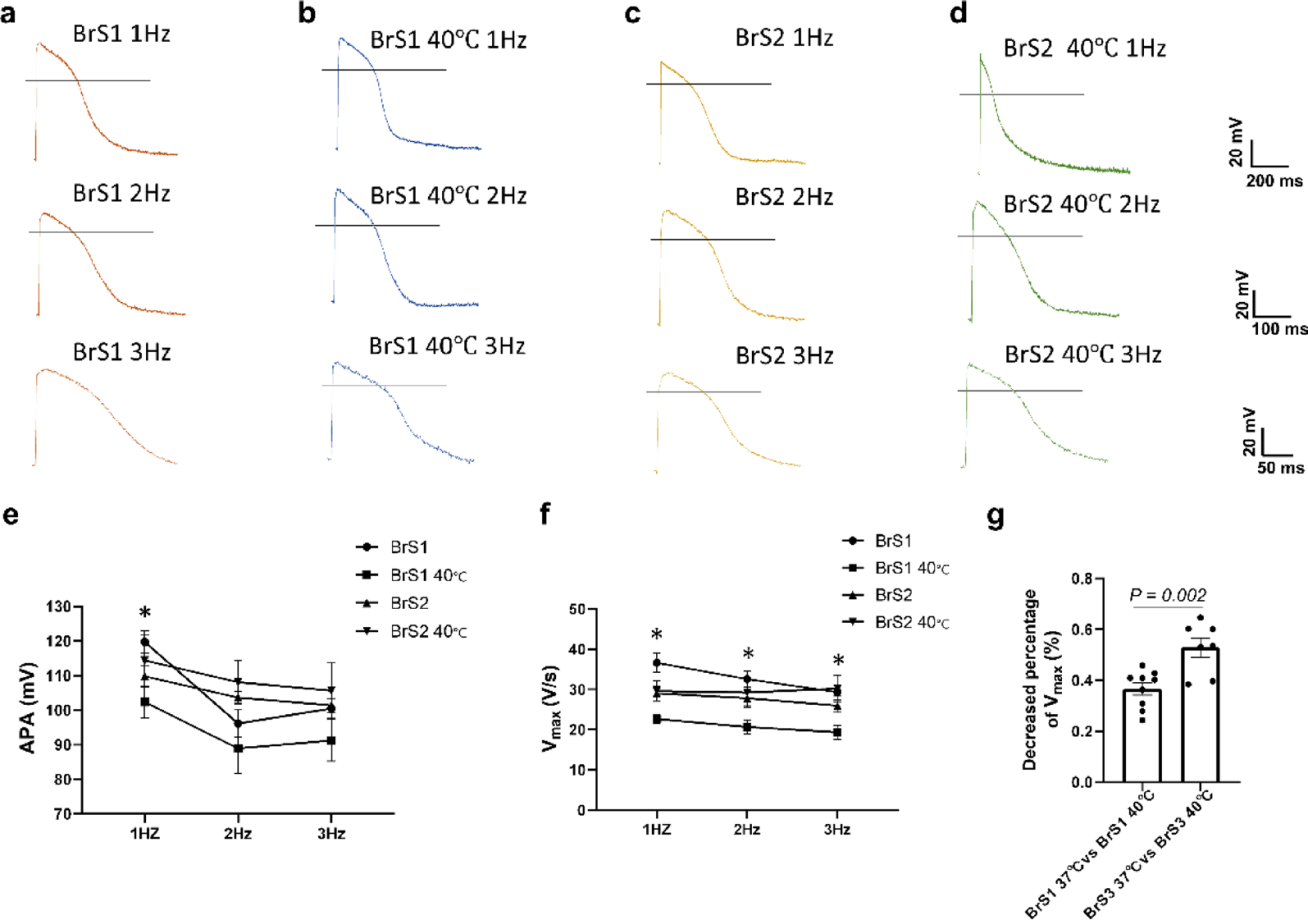
Changes of action potential in BrS cell lines with hyperthermia treatment. Action potentials were measured in hiPSC-CMs from the BrS-patients at 37℃ (BrS1 and BrS2) and 40℃ for 24 h (BrS1 40℃ and BrS2 40℃) at 1 Hz, 2 Hz and 3 Hz. (**a**–**d**) Representative traces of action potentials of each group. (**e**) Mean values of the amplitude (APA) of APs at 1–3 Hz in each group. (**f**) Mean values of maximal depolarization velocity (V_max_) of APs at 1–3 Hz in each group. (**g**) Decreased percentage of V_max_ of APs at 1 Hz between BrS1 and BrS3. The p values are determined by One way-ANOVA with Holm-Sidak post-test (**e**, **f**) or the unpaired Student’s t-test (**g**). *p < 0.05 BrS1 versus BrS1 40℃

The characteristics of action potentials, including action potential amplitude (APA), the resting potential (RP), the maximum depolarization velocity (V_max_), the action potential duration at 50% (APD 50) and 90% (APD 90) repolarization were measured in BrS1 and BrS2 at 1 Hz (figure S3 a-e), 2 Hz (figure S3 f-j), and 3 Hz (figure S3 k-o). APAs were significantly reduced in BrS2 at 1 Hz and in BrS1 at 2 Hz (figure S3 a, f). The V_max_ were significantly decreased in BrS1 and BrS2 compared to WT cell line at 1, 2 and 3 Hz (figure S3 b, g, i). All these changes in BrS cell lines were rescued in isogenic cell lines, respectively (figure S3). The representative traces of action potentials of each group were displayed in Fig. 1a, c and figure S4 a-c. The changes of APs in BrS1 and BrS2 hiPSC-CMs are similar to that observed in BrS3 cells (Table 1).

**Table 1:** The electrophysiological characteristics of action potential in different cell lines

	BrS1	Iso1	BrS2	Iso2	BrS3	Iso3
APA (mV)	119.71 ± 3.29	123.70 ± 2.52	109.79 ± 3.13	125.20 ± 4.44	114.54 ± 5.85	141.22 ± 4.69
V_max_ (V/s)	36.61 ± 2.35	61.74 ± 4.79	29.02 ± 1.94	61.12 ± 7.75	41.06 ± 1.08	78.74 ± 1.05
APD50 (ms)	183.15 ± 19.36	239.76 ± 36.34	192.99 ± 36.31	186.54 ± 14.21	144.60 ± 20.41	126.67 ± 19.64
APD90 (ms)	304.51 ± 12.73	332.07 ± 38.26	377.47 ± 32.09	277.03 ± 37.83	257.45 ± 29.03	234.44 ± 36.45
RP (mV)	-72.65 ± 1.09	-74.33 ± 3.00	-74.73 ± 0.96	-76.04 ± 1.23	-73.23 ± 1.46	-74.54 ± 0.98

The data were presented by mean ± SEM

*BrS1* SCN10A-hiPSC-CMs, Iso1 isogenic cells of SCN10A-hiPSC-CMs, *BrS2* CACNB2-hiPSC-CMs, *Iso2* isogenic cells of CACNB2-hiPSC-CMs, *BrS3* SCN5A-hiPSC-CMs, Iso3 isogenic cells of SCN5A-hiPSC-CMs, *APA* action potential amplitude, V_max_ the maximal velocity of depolarization, *APD50* action potential duration at 50% repolarization, *APD90* action potential duration at 90% repolarization, RP resting potential

**Fig. 2:**
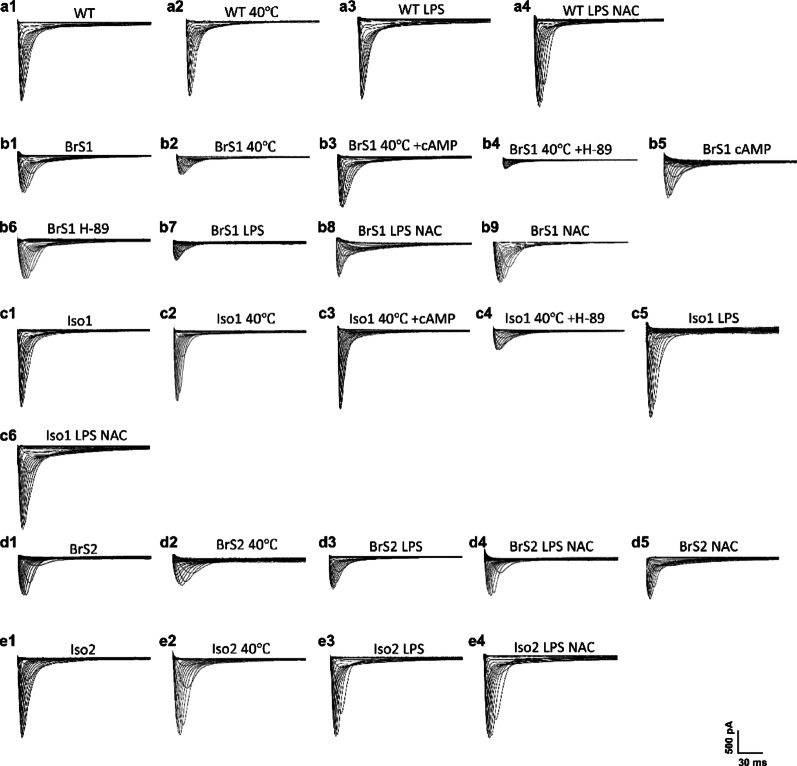
Representative traces of peak sodium channel currents (I_Na_) of WT, BrS and isogenic cell lines. Peak I_Na_ was measured with or without hyperthermia, cAMP, H-89, LPS or NAC treatment in hiPSC-CMs from a healthy donor (WT), a BrS-patient with the variant in *SCN10A* (BrS1), a BrS-patient with the variant in *CACNB2* (BrS2) and the variant-corrected cell lines (Iso1 and Iso2). (a1-a4) Representative traces of peak I_Na_ of WT. (b1-b9) Representative traces of peak I_Na_ of BrS1. (c1-c6) Representative traces of peak I_Na_ of Iso1. (d1-d5) Representative traces of peak I_Na_ of BrS2. (e1-e4) Representative traces of peak I_Na_ of Iso2

Consistent with the reduction of V_max_ of action potentials in BrS cell lines, the peak I_Na_ of BrS1 and BrS2 were decreased compared to the WT and isogenic cell lines (figure S5 a-b). In addition, activation curves of I_Na_ of BrS2 were shifted to more positive potentials and inactivation curves of I_Na_ of BrS1 were shifted to more negative potentials (figure S5 c-f). The representative traces of peak I_Na_ of each group were displayed in Fig. 2a1, b1, c1, d1, e1. The changes of I_Na_ in BrS1 and BrS2 hiPSC-CMs are similar to that observed in BrS3 cells (Table 2).

**Table 2:** The electrophysiological characteristics of peak I_Na_ in different cell lines

	BrS1	Iso1	BrS2	Iso2	BrS3	Iso3
Peak I_Na_ (pA/pF)	-32.59 ± 5.65	-76.61 ± 11.66	-45.64 ± 11.33	-98.26 ± 11.88	-41.54 ± 1.74	-100.00 ± 13.52
Activation (V_0.5_, mV)	-47.00 ± 1.85	-45.63 ± 1.65	-44.50 ± 2.44	-51.55 ± 1.39	-37.84 ± 0.62	-41.06 ± 1.43
Inactivation (V_0.5_, mV)	-82.72 ± 2.02	-76.75 ± 0.79	-72.60 ± 2.16	-79.89 ± 2.34	-77.98 ± 1.25	-76.34 ± 0.86
Recovery (Tau, ms)	11.09 ± 1.37	10.47 ± 1.05	8.55 ± 0.91	14.30 ± 3.20	20.71 ± 1.37	14.60 ± 1.78

The data were presented by mean ± SEM

*BrS1* SCN10A-hiPSC-CMs, Iso1 isogenic cells of SCN10A-hiPSC-CMs, *BrS2* CACNB2-hiPSC-CMs, *Iso2* isogenic cells of CACNB2-hiPSC-CMs, *BrS3* SCN5A-hiPSC-CMs, *Iso3* isogenic cells of SCN5A-hiPSC-CMs, *I*_*Na*_ peak sodium current, *V*_*0*.5_ the voltage at which the conductance was half-maximal, *Tau* the time constant of channel recovery

### The effect of hyperthermia on action potentials of BrS cell lines

To explore the effect of high temperature on BrS cell lines, the characteristics of action potentials were measured after 24 h cell culture in 40 °C mimicking fever. Increasing the culture temperature from 37 °C to 40 °C in BrS1 reduced significantly the V_max_ and slightly reduced APA at 1–3 Hz (Fig. 1a-b, e-f). However, high temperature showed no significant effect on V_max_ or APA of BrS2 cells (Fig. 1a-f), although it shortened the APD50 in BrS2 at 1 Hz (figure S6b). Moreover, high temperature also decreased the V_max_ of AP in BrS3 cell line (figure S7d). Although the V_max_ of BrS1 and BrS3 cell lines were both reduced by hyperthermia, high temperature showed stronger effect on V_max_ in BrS3 (Fig. 1g). The representative traces of action potentials of BrS lines with hyperthermia were displayed in Fig. 1a-d and S8a (for BrS3).

### The effect of hyperthermia on I_Na_ and arrhythmogeneity of BrS cell lines and the role of protein kinase A

**Fig. 3:**
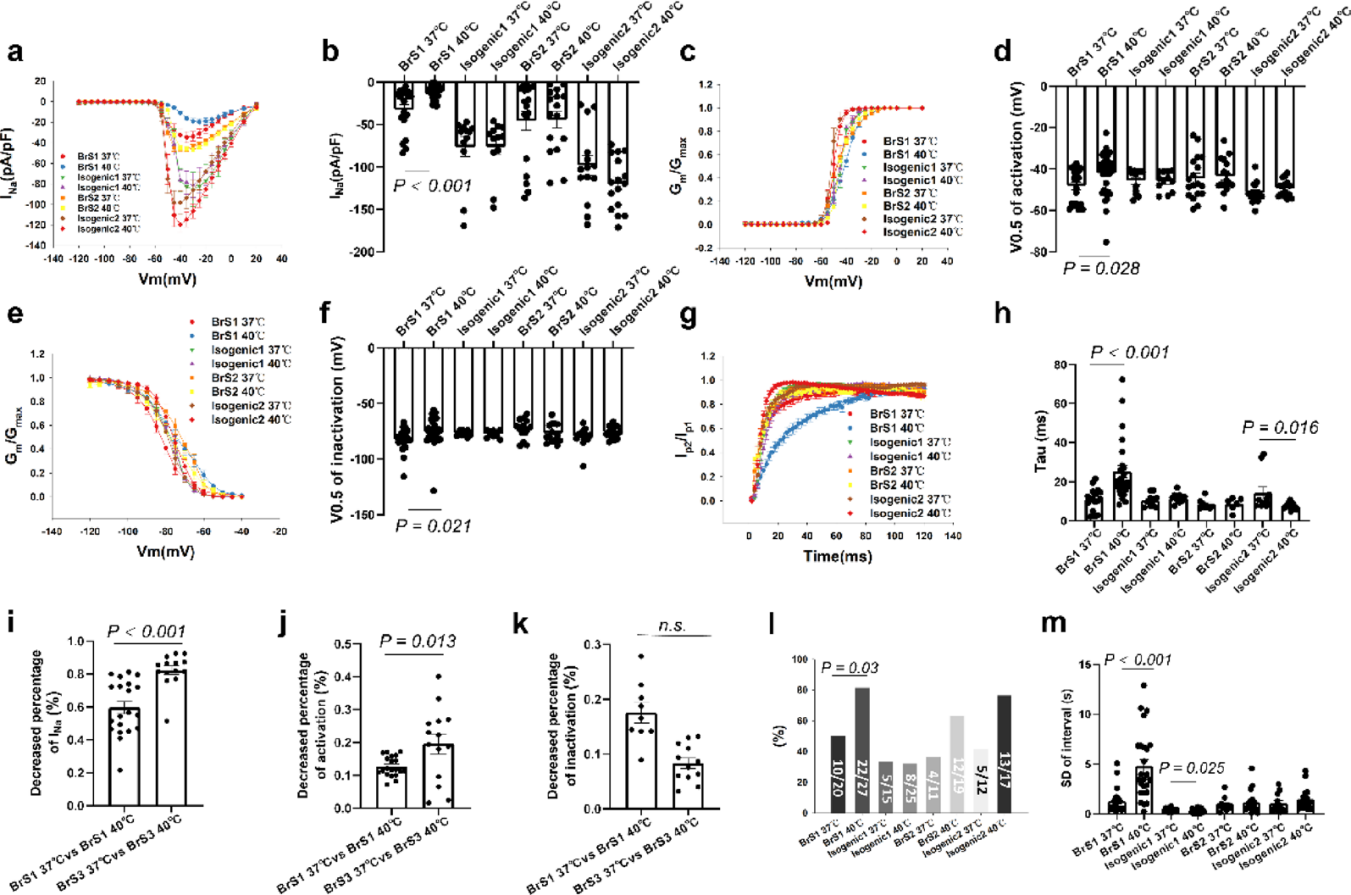
Hyperthermia further exacerbated the loss-of-function of sodium channels in BrS1 cell line. Peak sodium channel currents (I_Na_) were measured in hiPSC-CMs from the BrS-patients (BrS1 and BrS2) and the variant-corrected cell lines (Isogenic1 and Isogenic2) at 37℃ and 40℃ for 24 h (BrS1 40℃ and BrS2 40℃; Isogenic1 40℃ and Isogenic2 40℃). (**a**) Current-voltage (I-V) relationship curves of peak I_Na_ in each group. (**b**) Mean values of peak I_Na_ at -40 mV in each group. (**c**) Activation curves of peak I_Na_ in each group. (**d**) Mean values of potential at 50% activation (V0.5) in each group. (**e**) Inactivation curves of peak I_Na_ in each group. (**f**) Mean values of potential at 50% inactivation (V0.5) in each group. (**g**) Recovery curves of peak I_Na_ in each group. (**h**) Mean values of time constant (Tau) of recovery from inactivation in each group. (**i**) Decreased percentage of I_Na_ at -40 mV between BrS1 and BrS3. (**j**) Decreased percentage of V0.5 of activation (V0.5) between BrS1 and BrS3. (**k**) Decreased percentage of V0.5 of inactivation (V0.5) between BrS1 and BrS3. (**l**) The percentage of cells showing arrhythmic events (EAD-like events or triggered events) in each group. (**m**) Mean values of interval variability in each group. The p values are determined by One way-ANOVA with Holm-Sidak post-test (c, d, f, h, m), the unpaired Student’s t-test (**i**–**k**) or Fisher-test (l)

Consistent with the reduction of V_max_ of action potentials in BrS1 cell line, the peak I_Na_ of BrS1 was decreased after culture in 40 °C (Fig. 3a-b). The activation curves and inactivation curves of I_Na_ of BrS1 with hyperthermia treatment were shifted to more positive potentials (Fig. 3c-f). The recovery from inactivation of I_Na_ in BrS1 with hyperthermia was decelerated (Fig. 3g-h). Hyperthermia failed to influence I_Na_ in BrS2 or isogenic control cells (Fig. 3). The high temperature showed a more significant impact on the I_Na_ in BrS3 (Fig. 3i), along with a shift towards more positive potentials in activation curves and a less shift of inactivation curves (Fig. 3j-k). The representative traces of peak I_Na_ of BrS lines with hyperthermia were displayed in Fig. 2b1-b2 (BrS1), c1-c2 (Iso1), d1-d2 (BrS2), e1-e2 (Iso2).

Ca^2+^ is closely associated with contraction of cardiomyocytes. Abnormal Ca^2+^ handling can cause arrhythmogenic events, including the delayed afterpotential (DAD), the early afterdepolarization (EAD), and so on. The calcium current can be allowed to recover from inactivation because of the abnormal prolongation of APD, and the raised calcium current can cause a second upstroke from plateau of APs, leading to EAD [24]. EAD is thought to be involved in arrhythmias associated with Q-T prolongation. On the other hand, abnormal calcium influx or release after repolarization of APs can cause extra depolarizations, so called DAD. When a DAD reaches the threshold of activation, it can trigger an extra beat and hence DADs may also cause arrhythmias. It has been shown that calcium transient measurements could detect arrhythmia-like evets in hiPSC-CMs derived from BrS-patients [25]. Therefore, we defined the arrhythmia-like events as the presence of EAD-like or DAD-like or triggered beat events, and detected them in hiPSC-CMs by using the calcium transient measurement. Our results showed that BrS1 cells with hyperthermia more frequently displayed arrhythmia-like events and showed higher interval variability compared to cells in 37 °C (Fig. 2l-m). However, hyperthermia showed no significant effect on I_Na_ and arrhythmogeneity of WT, BrS2, and isogenic2 cell lines (Fig. 3, figure S8). In BrS3 cells, hyperthermia also increased arrhythmic events and the interval variability (figure S9). The representative traces of spontaneous calcium transients of each group with hyperthermia were displayed in figure S10 a1-a2 (WT), b1-b2 (BrS1), c1-c2 (Iso1), d1-d2 (BrS2), e1-e2 (Iso2), f1-f2 (BrS3).

Since *CACNB2* encodes the β subunit of voltage-gated calcium channels Cav1.2, we also tested the effect of hyperthermia on I_Ca−L_ in BrS2 cell line. The BrS2 displayed lower I_Ca−L_ compared to WT and isogenic cell lines, but hyperthermia showed no significant effect on I_Ca−L_ of WT, BrS2 and isogenic cell lines (figure S11).

**Fig. 4:**
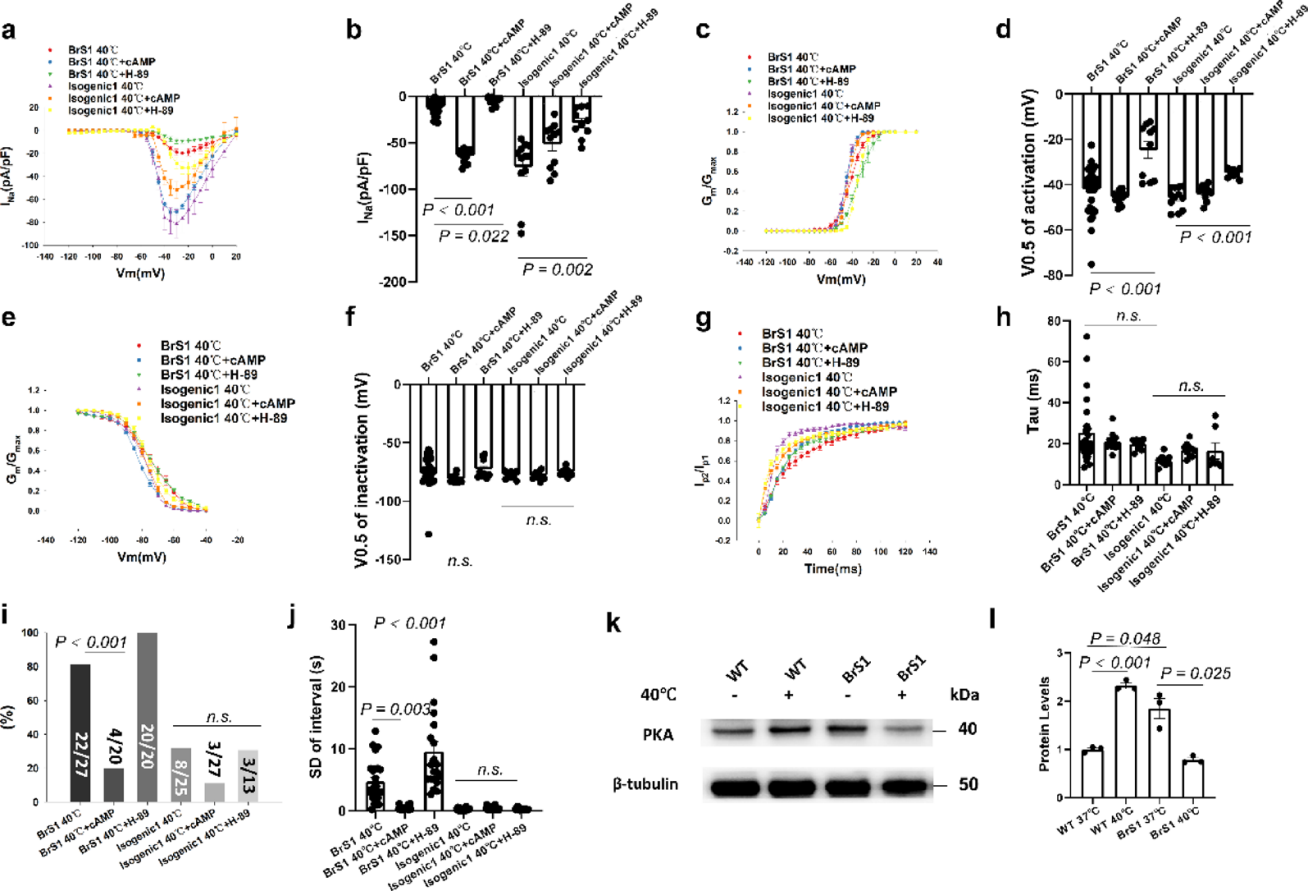
The effect of hyperthermia on BrS1 cells was mediated by PKA signaling. Peak sodium channel currents (I_Na_) were measured in hiPSC-CMs from the BrS-patient with the variant in *SCN10A* (BrS1) and the variant-corrected cell line (Isogenic1) at 37℃ and 40℃ for 24 h and 40℃ with PKA inhibitor (H-89, 10µM, 24 h) or activator (8-Bromo-cAMP, 5µM, 24 h). (**a**) Current-voltage (I-V) relationship curves of peak I_Na_ in each group. (**b**) Mean values of peak I_Na_ at -40 mV in each group. (c) Activation curves of peak I_Na_ in each group. (**d**) Mean values of potential at 50% activation (V0.5) in each group. (**e**) Inactivation curves of peak I_Na_ in each group. (**f**) Mean values of potential at 50% inactivation (V0.5) in each group. (**g**) Recovery curves of peak I_Na_ in each group. (**h**) Mean values of time constant (Tau) of recovery from inactivation in each group. (**i**) The percentage of cells showing arrhythmic events (EAD-like events or triggered events) in each group. (**j**) Mean values of interval variability in each group. (**k**) and (**l**) Representative (**k**) and statistical data (**l**) of western blot in BrS1, *n* = 3 (number of independent experiments). Full-length blots are presented in Supplementary Western blots. The p values are determined by One way-ANOVA with Holm-Sidak post-test (b, d, f, h, j, l) or Fisher-test (**i**)

To further explore the underlying mechanism of effect of hyperthermia on peak I_Na_ and arrhythmogeneity in BrS cells, the protein kinase A (PKA) activator (8-Bromo-cAMP, 5 µM, 24 h) and inhibitor (H-89, 10 µM, 24 h) were utilized. The cAMP treatment increased the I_Na_ in BrS1 but not isogenic1 cell line. H-89 further reduced I_Na_ in BrS1 (Fig. 4a-b). H-89 but not cAMP enhanced hyperthermia effect on channel activation without significant effect on inactivation and recovery of the channel in BrS1 (Fig. 4c-h). Although the H-89 effects on peak I_Na_ and activation were detected also in isogenic1 cells, the effects were smaller than that in BrS1 cells (Fig. 4). In addition, cAMP treatment improved and H-89 treatment exacerbated the peak I_Na_ reduction and arrhythmogeneity of BrS1 cells compared to the cells with hyperthermia alone, indicating that PKA may play a role in this process (Fig. 4a-j). The PKA expression was reduced in BrS1 and increased in WT after hyperthermia treatment (Fig. 4k, l). Furthermore, at 37 °C, cAMP or H-89 treatment alone showed no significant on I_Na_ (figure S12), The representative traces of peak I_Na_ of each group were displayed in Fig. 2b1-b6 (BrS1), c2-c4 (Iso1). In BrS 3 cell line, cAMP attenuated and H-89 exacerbated the arrhythmogeneity (figure S9). The representative traces of spontaneous calcium transients of each group were displayed in figure S10 a1-a2 (WT), b1-b6 (BrS1), c2-c4 (Iso1), f1-f4 (BrS3). The distinct effects of hyperthermia on electrophysiological characteristics in different BrS cell lines (BrS1, 2 and 3) were displayed in Table S1.

### The effect of LPS on action potentials of BrS cell lines

**Fig. 5:**
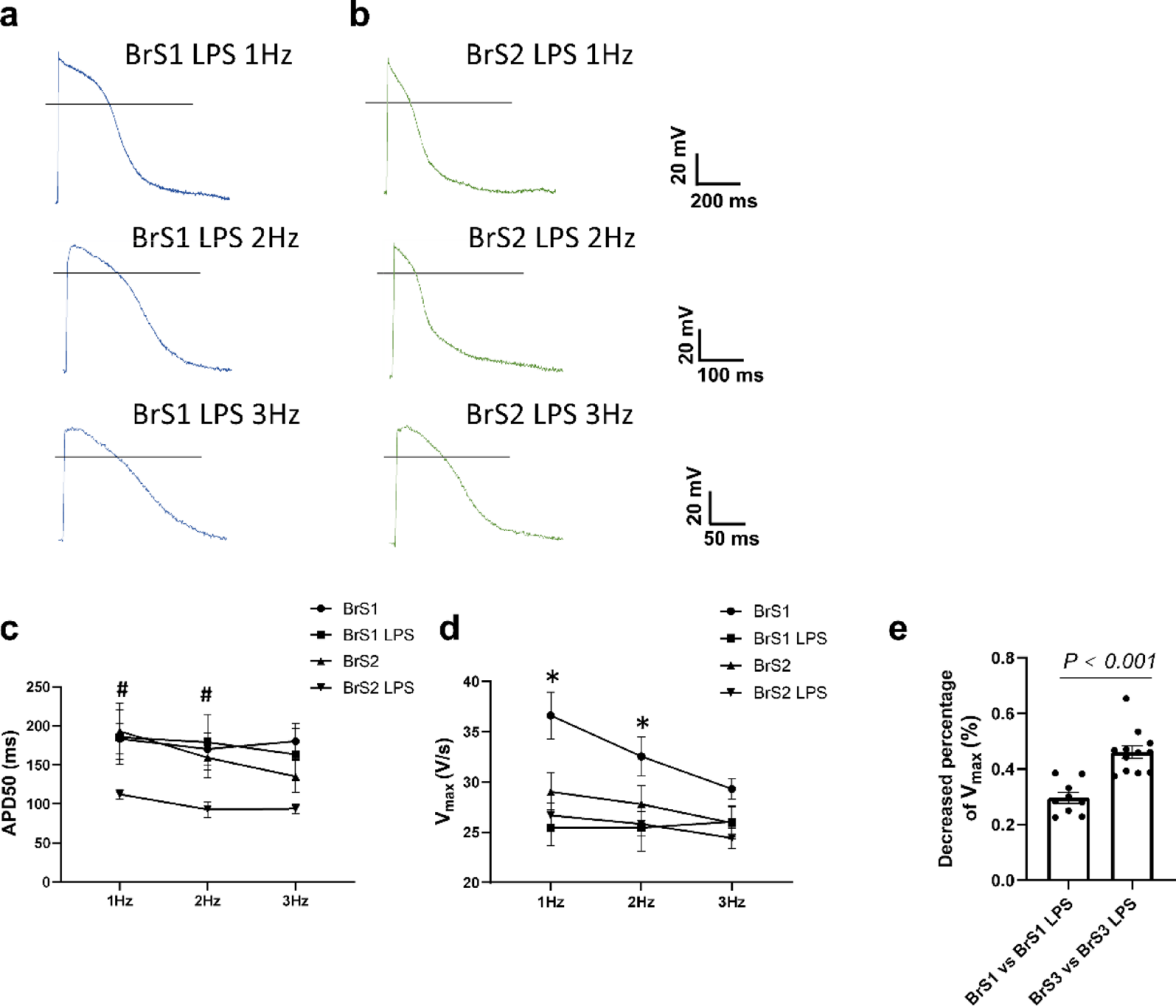
Changes of action potential in BrS hiPSC-CMs treated with LPS. Action potentials were measured in hiPSC-CMs from the BrS-patients at 37℃ (BrS1 and BrS2) and 37℃ with LPS treatment (2 µg/ml, 24 h) (BrS1 LPS and BrS2 LPS) at 1 Hz, 2 Hz and 3 Hz. (**a**,** b**) Representative traces of action potentials of each group. (c) Mean values of the APD50 of APs at 1–3 Hz in each group. (d) Mean values of maximal depolarization velocity (V_max_) of APs at 1–3 Hz in each group. (e) Decreased percentage of V_max_ of APs at 1 Hz between BrS1 and BrS3. The p values are determined by One way-ANOVA with Holm-Sidak post-test (**c**, **d**) or the unpaired Student’s t-test (**e**). *p < 0.05 BrS1 versus BrS1 40℃; ^**#**^*p* < 0.05 BrS2 versus BrS2 LPS

After treatment of LPS (2 µg/ml, 24 h) mimicking infection, the V_max_ of action potentials at 1 and 2 Hz was reduced in BrS1 (Fig. 5a-d), and the APD50 of action potentials at 1 and 2 Hz was shortened in BrS2 (Fig. 5a-d). However, LPS treatment displayed no effect on other characteristics of action potentials (figure S13). LPS showed a more significant effect on the V_max_ in BrS3 compared to BrS1 (Fig. 5e). The representative traces of action potentials of BrS lines with LPS were displayed in Figs. 1a, c and 5a-b.

### The effect of LPS on peak I_Na_ and arrhythmogeneity of BrS cell lines and the role of ROS

**Fig. 6:**
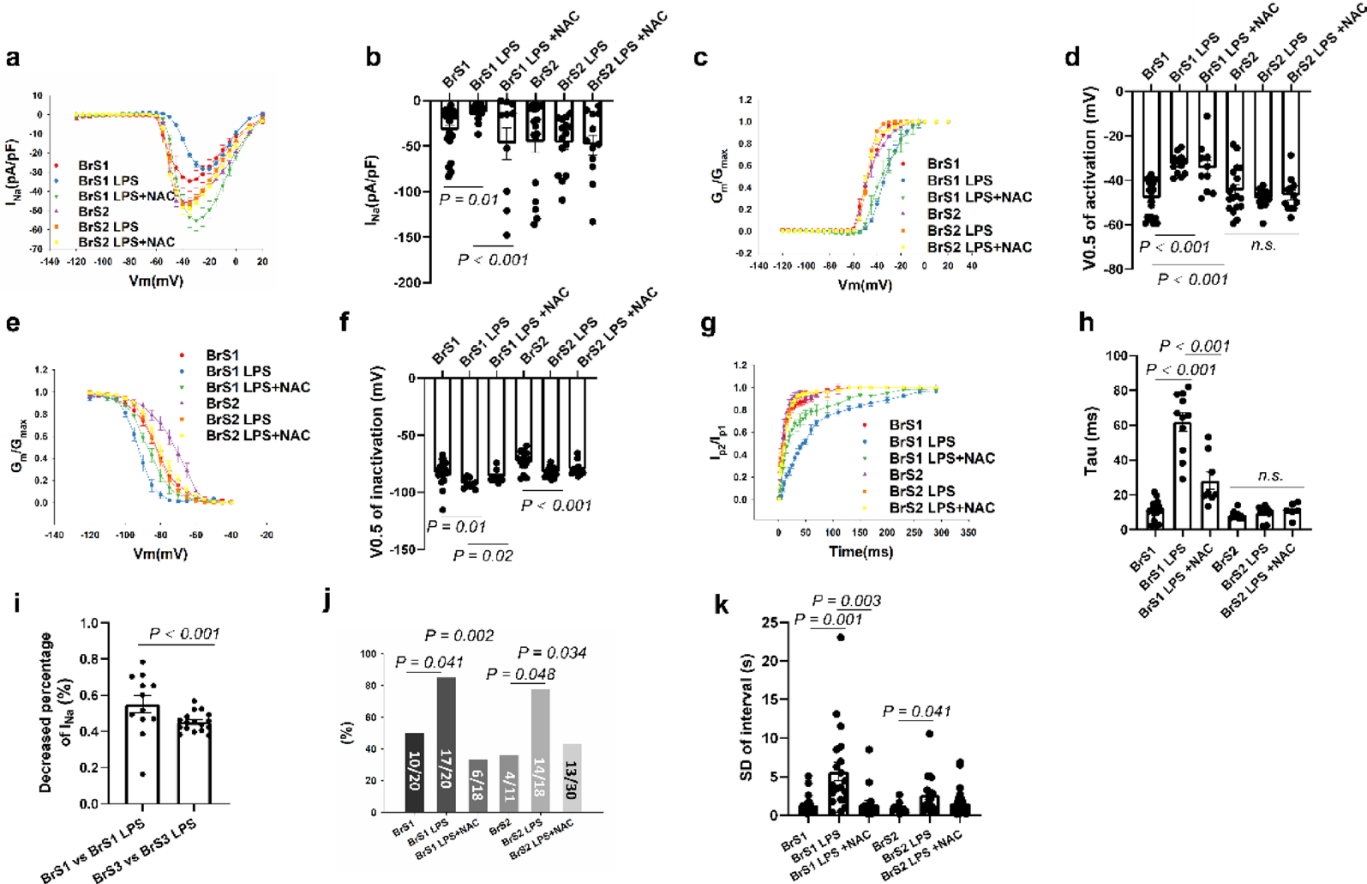
LPS further exacerbated the loss-of-function of sodium channels in BrS1 cell line. Peak sodium channel currents (I_Na_) were measured in hiPSC-CMs from the BrS-patients at room temperature (BrS1 and BrS2) and at room temperature in presence of LPS (2 µg/ml, 24 h, BrS1 LPS, BrS2 LPS) and LPS plus ROS blocker (NAC, 1 mM, 24 h, BrS1 LPS + NAC, BrS2 LPS + NAC). (**a**) Current-voltage (I-V) relationship curves of peak I_Na_ in each group. (**b**) Mean values of peak I_Na_ at -40 mV in each group. (**c**) Activation curves of peak I_Na_ in each group. (**d**) Mean values of potential at 50% activation (V0.5) in each group. (**e**) Inactivation curves of peak I_Na_ in each group. (**f**) Mean values of potential at 50% inactivation (V0.5) in each group. (**g**) Recovery curves of peak I_Na_ in each group. (**h**) Mean values of time constant (Tau) of recovery from inactivation in each group. (**i**) Decreased percentage of I_Na_ at -40 mV between BrS1 and BrS3. (**j**) The percentage of cells showing arrhythmic events (EAD-like events or triggered events) in each group. k Mean values of interval variability in each group. The p values are determined by One way-ANOVA with Holm-Sidak post-test (b, d, f, h, k), the unpaired Student’s t-test (**i**) or Fisher-test (**j**)

LPS treatment increased ROS generation in BrS1 and BrS2 cell lines (figure S14). Consistent with the reduced V_max_ in BrS1 but not BrS2 cell line with LPS treatment, the peak I_Na_ was further reduced after LPS treatment, and ROS blocker (NAC, 1 mM, 24 h) rescued the effect of LPS on I_Na_ (Fig. 6a-b), indicating ROS play an important role in LPS mediated I_Na_ reduction. Moreover, the activation curve of I_Na_ of BrS1 was shifted to more positive potentials after LPS treatment (Fig. 6c-d). Inactivation curve was shifted to more negative potentials and recovery curves from inactivation were decelerated after LPS treatment, which could be rescued by using NAC (Fig. 6e-h). In addition, the inactivation curve of peak I_Na_ in BrS2 was also shifted to more negative potentials after LPS treatment (Fig. 6e-f). Interestingly, LPS showed a more significant effect on the I_Na_ in BrS1 than BrS3 cells at -40mV, which may be caused by the effect of LPS on sodium channel kinetics in BrS1 but not in BrS3 (Fig. 6i). Moreover, NAC treatment alone showed no significant effects on I_Na,_ arrhythmia-like events and interval variability in both BrS1 and BrS2 without LPS challenge (figure S15). The representative traces of peak I_Na_ of hiPSC-CMs with LPS treatment were displayed in Fig. 2 (a1, a3, a4 for WT; b1, b7-b9 for BrS1; c1, c5, c6 for Iso1; d1, d3-d5 for BrS2; e1, e3, e4 for Iso2).

Both BrS1 and BrS2 cells displayed more arrhythmia-like events and higher interval variability after LPS treatment (Fig. 6j, k). LPS treatment also increased interval variability in WT cell line (figure S16j), whereas other characteristics were not changed in WT and other isogenic cell lines (figure S16). In BrS3 cells, arrhythmic events were increased significantly by LPS, while the percentage of cells showing arrhythmic events and SD of interval were only slightly changed (figure S9). The representative traces of calcium transients of hiPSC-CMs with LPS treatment were displayed in S11 (a1, a3, a4 for WT; b1, b7-b9 for BrS1; c1, c5, c6 for Iso1; d1, d3-d5 for BrS2; e1, e3, e4 for Iso2; f1, f5, f6 for BrS3).

### The effect of LPS on I_Ca−L_ of BrS cells with *CACNB2* variant

**Fig. 7:**
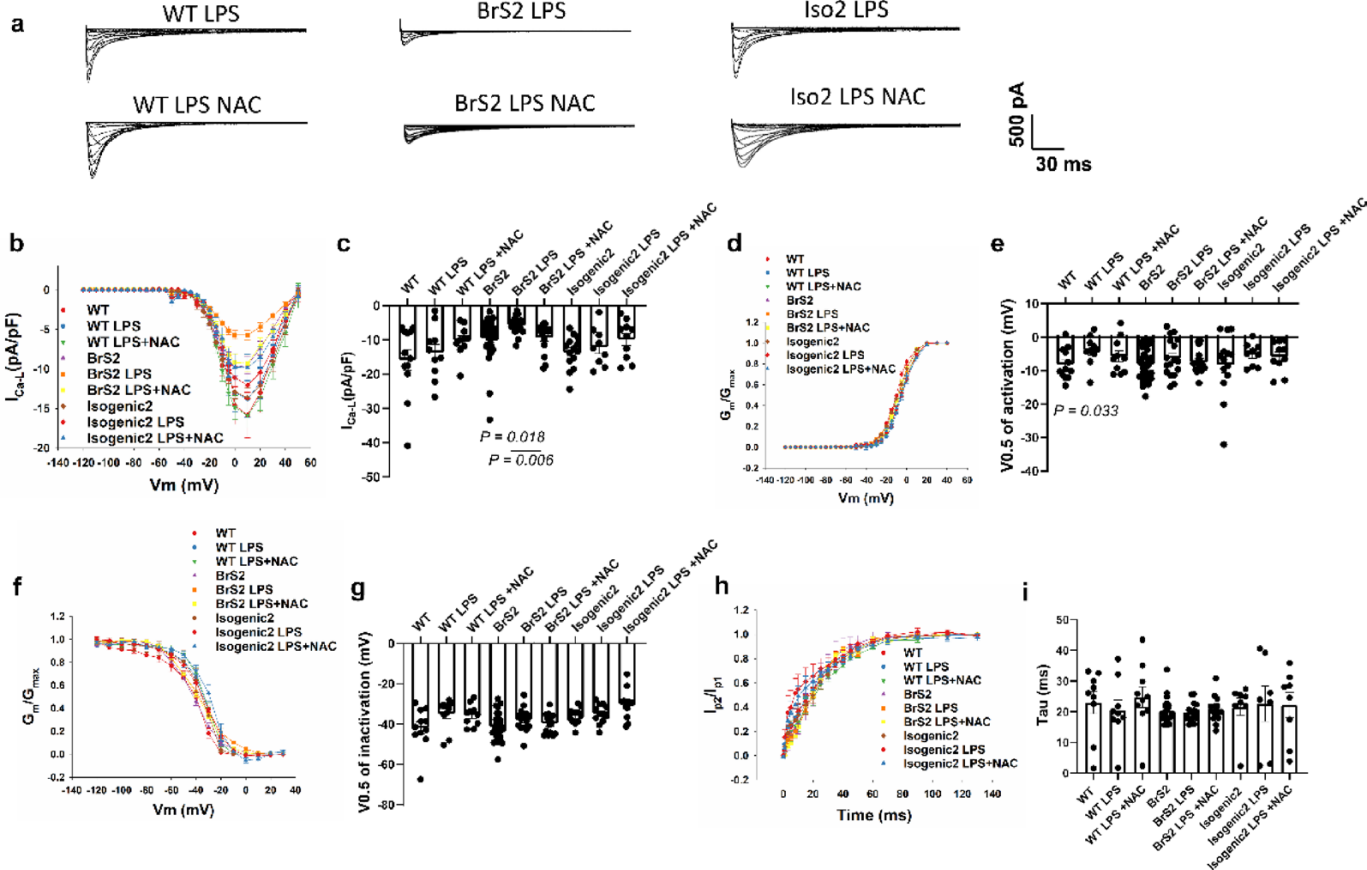
LPS further exacerbated the loss-of-function of calcium channels in BrS2 cell line. L-type calcium channel currents (I_Ca-L_) were measured at room temperature in hiPSC-CMs from a healthy donor (WT), the BrS-patient with the variant in *CACNB2* (BrS2) and the variant-corrected cell line (Isogenic2), and at room temperature in presence of LPS (2 µg/ml, 24 h, WT LPS, BrS2 LPS, Isogenic2 LPS) or LPS plus ROS blocker (NAC, 1 mM, 24 h, WT LPS + NAC, BrS2 LPS + NAC, Isogenic2 LPS + NAC). (**a**) Representative traces of L-type calcium channel currents (I_Ca-L_) of each group. (**b**) Current-voltage (I-V) relationship curves of I_Ca-L_ in each group. (**c**) Mean values of I_Ca-L_ at 10 mV in each group. (**d**) Activation curves of I_Ca-L_ in each group. (**e**) Mean values of potential at 50% activation (V0.5) in each group. (**f**) Inactivation curves of I_Ca-L_ in each group. (**g**) Mean values of potential at 50% inactivation (V0.5) in each group. (**h**) Recovery curves of I_Ca-L_ in each group. (**i**) Mean values of time constant (Tau) of recovery from inactivation in each group. The p values are determined by One way-ANOVA with Holm-Sidak post-test

The I_Ca−L_ of BrS2 was reduced after LPS treatment, which was rescued by using NAC, indicating LPS affected the I_Ca−L_ through ROS signaling in BrS cells with *CACNB2* variant (Fig. 7a-c). The activation curves of I_Ca−L_ were shifted to more positive potentials after LPS treatment, and this effect could be partially reversed by NAC (Fig. 7d, e). The inactivation and recovery curves were not changed after LPS treatment.

### The effect of LPS on arrhythmogeneity of BrS cells was mediated by ROS/IL-6 signaling

**Fig. 8:**
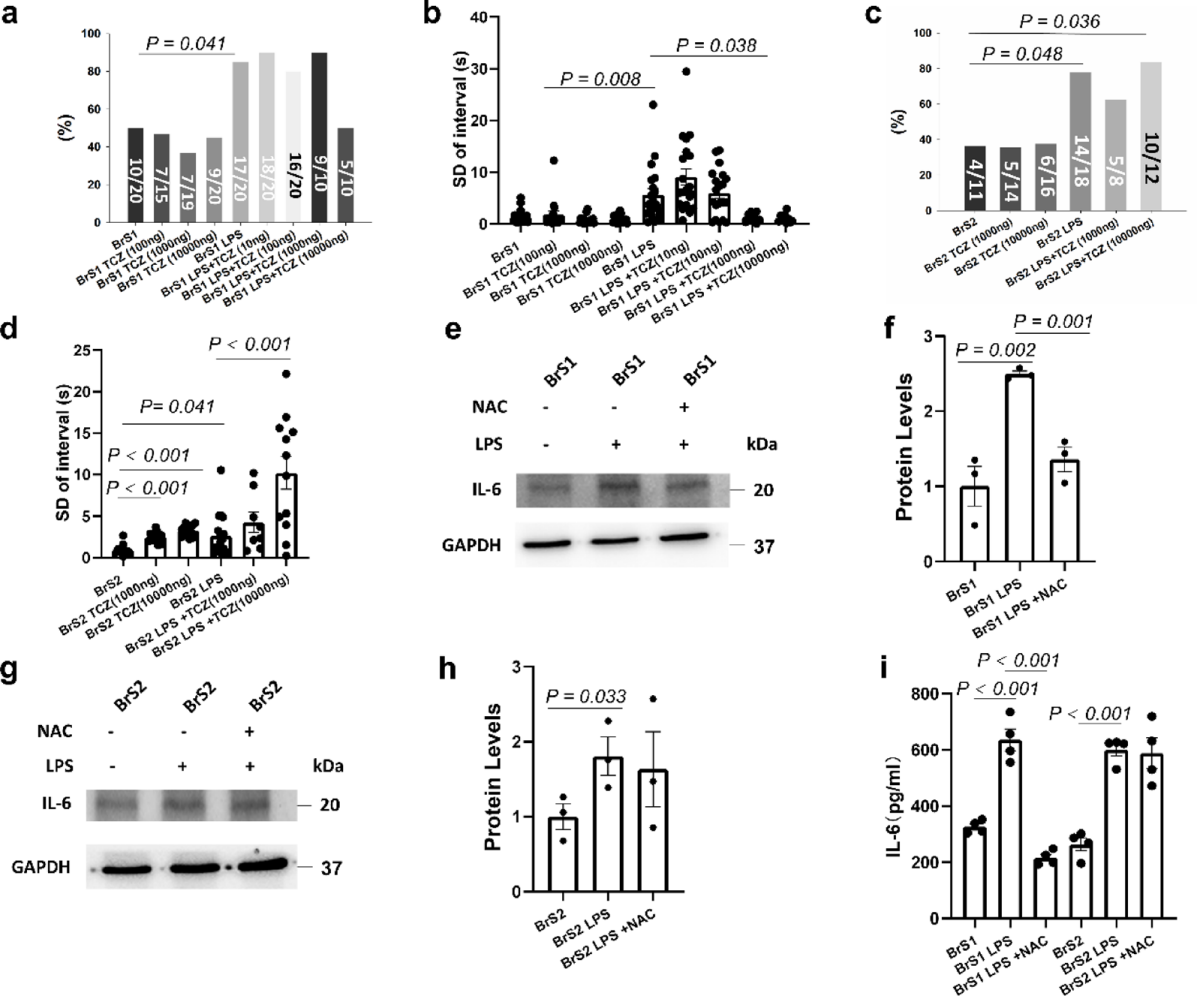
The effect of LPS on arrhythmogeneity of BrS1 cells was mediated by ROS/IL-6 signaling. The arrhythmogeneity was measured by calcium transients at room temperature in hiPSC-CMs from the two BrS-patients (BrS1 and BrS2) in presence of LPS (2 µg/ml, 24 h, BrS1 LPS, BrS2 LPS) or LPS plus IL-6 receptor inhibitor tocilizumab (TCZ, 10-10000 ng/ml, 24 h) (BrS1 LPS + TCZ, BrS2 LPS + TCZ) or tocilizumab alone (BrS1 TCZ 1000 ng and BrS2 TCZ 1000 ng). The expression of IL-6 was measured by western blot (e-h) and Elisa (i) in BrS cell lines in absence (BrS1 and BrS2) and presence of LPS (2 µg/ml, 24 h, BrS1 LPS, BrS2 LPS) or LPS plus ROS blocker (NAC, 1 mM, 24 h, BrS1 LPS + NAC, BrS2 LPS + NAC). (**a**) The percentage of cells showing arrhythmic events (EAD-like events or triggered events) in each group of BrS1. (**b**) Mean values of interval variability in each group of BrS1. (**c**) The percentage of cells showing arrhythmic events (EAD-like events or triggered events) in each group of BrS2. (**d**) Mean values of interval variability in each group of BrS2. (**e**) and (**f**) Representative (**e**) and statistical data (**f**) of western blot in BrS1 cells, *n* = 3 (number of independent experiments). (**g**) and (**h**) Representative (**g**) and statistical data (**h**) of western blot in BrS2 cells, *n* = 3 (number of independent experiments). Full-length blots are presented in Supplementary Western blots. (**i**) The concentration of IL-6 in cell culture medium in each group. The p values are determined by One way-ANOVA with Holm-Sidak post-test (b, d, f, h, i) or Fischer-test (a, c)

**Fig. 9:**
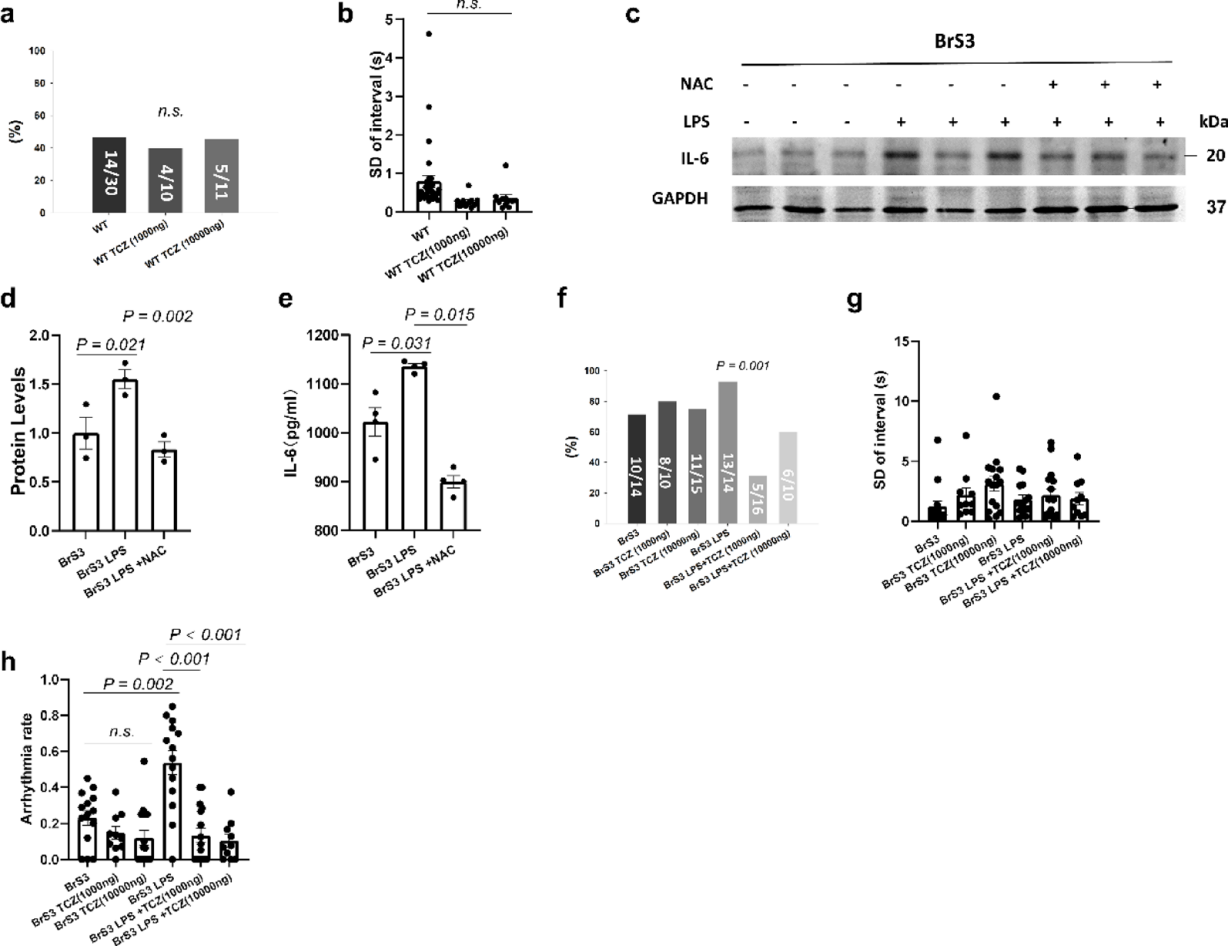
The effect of IL-6 on arrhythmogeneity of WT and BrS3 cells. The arrhythmogeneity was measured by calcium transients at room temperature in WT and BrS3 cells in presence of LPS (2 µg/ml, 24 h) or LPS plus IL-6 receptor inhibitor tocilizumab (TCZ, 1000–10000 ng/ml, 24 h) or tocilizumab alone (TCZ, 1000–10000 ng/ml, 24 h).The expression of IL-6 was measured by western blot (**c**,** d**) and Elisa (**e**) in BrS3 cell line in absence and presence of LPS (2 µg/ml, 24 h) or LPS plus oxidants blocker (NAC, 1 mM, 24 h). (**a**) The percentage of cells showing arrhythmic events (EAD-like events or triggered events) in each group. (**b**) Mean values of interval variability in each group. (**c**) and (**d**) Representative (**c**) and statistical data (**d**) of western blot in BrS3 cells, *n* = 3 (number of independent experiments). Full-length blots are presented in Supplementary Western blots. (**e**) The concentration of IL-6 in cell culture medium in each group. (**f**) The percentage of cells showing arrhythmic events (EAD-like events or triggered events) in each group. (**g**) Mean values of interval variability in each group. (**h**) Arrhythmia event rate in 100 s in each group. The p values are determined by One way-ANOVA with Holm-Sidak post-test (a, b, d, e, g, h) or Fischer-test (f)

To further explore the mechanisms of the effect of LPS on arrhythmogeneity of BrS cells, the blocker of IL-6 receptor tocilizumab (TCZ, 10-10000ng/ml, 24 h) was utilized. TCZ treatment alone showed no effect on arrhythmogeneity of BrS1 cells, whereas it could rescue the effect of LPS in a high concentration (1000-10000ng/ml, 24 h) (Fig. 8a-b). However, TCZ alone or with LPS increased arrhythmia-like events and interval variability in BrS2 cells (Fig. 8c). TCZ treatment showed no or slight effect on WT cell line (Fig. 9a-b). The results of western blot and ELISA displayed that IL-6 levels were increased after LPS treatment, and IL-6 levels were decreased after NAC treatment in BrS1 but not BrS2 (Fig. 8e-i), indicating ROS effect was mediated by IL-6 in BrS1 but not BrS2.

In BrS 3, LPS increased arrhythmia-like events, NAC attenuated LPS effect (figure S9). LPS also increased IL-6 expression and production, which was blocked by NAC, and TCZ reduced LPS-induced arrhythmia-like events (Fig. 9c-h). The distinct effects of LPS on electrophysiological characteristics in different BrS cell lines (BrS1, 2 and 3) were displayed in Table S2.

### Phosphorylation level in BrS-hiPSC-CMs challenged by hyperthermia and LPS

To further explore mechanism underlying hyperthermia and LPS effects, we examined the total phosphorylation level of hiPS-CMs with and without challenge of hyperthermia or LPS. The total phosphorylation level in all BrS-hiPSC-CMs was lower than that in WT cells. Hyperthermia reduced the phosphorylation level in *SCN5A*- and *CACNB2*- but increased in *SCN10A*-cells **(figure S17)**. LPS reduced the phosphorylation level in *SCN5A*- and not *SCN10A*-cells **(figure S18)**.

### Hyperthermia reduced expression of beta1-receptor

Considering PKA expression was reduced by hyperthermia and adrenoceptor is important for PKA signaling, we checked the expression level in BrS-hiPSC-CMs challenged by hyperthermia. Interestingly, beta1-adrenoceptor expression was reduced by hyperthermia in both *SCN10A* and *SCN5A* cells but not in WT or isogenic cells (figure S17 c-f).

## Discussion

In the present study, our results displayed that high temperature and LPS challenge differentially exacerbated the loss-of-function of cardiac sodium channels and arrhythmogeneity of cardiomyocytes carrying *SCN10A*, *CACNB2* and *SCN5A* variants.

Although BrS is most often linked to *SCN5A* variants, increasing evidence highlights important roles of other genes [26]. Our recent studies demonstrated that hiPSC-CMs from a BrS patient with *SCN10A* variants showed markedly reduced peak sodium current (I_Na_) [16, 19], while hiPSC-CMs from a patient with a *CACNB2* variant displayed impaired L-type calcium currents (I_Ca−L_), shortened action potential duration (APD), and decreased depolarization velocity (V_max_), features of SQT syndrome overlapping with BrS [23]. The present study confirmed those findings, further supporting pathogenic roles of the variants.

Type 1 BrS ECG is frequently triggered during fever [27], and BrS patients with fever-induced type 1 BrS ECG have a higher risk of arrhythmic events, such as VF or sudden cardiac death (SCD) [28]. In a previous study, the *SCN5A*-Thr1620Met mutant in frog oocytes changed the sodium channel gating kinetics, rendering the channel more sensitive to fever [29]. Recent case reports showed that some BrS patients with COVID-19 infection were found to have the type-1 Brugada ECG pattern while maintaining febrile overnight. After treatment of antipyretic drugs, patients’ fever was resolved and ECG went back to normal [30, 31]. Although these studies did not investigate the underlying mechanisms, they did provide interesting information about the connection between genetic variants or genotypes and environmental stress. Our study showed that hyperthermia (40 °C) exacerbated the BrS phenotype by changing peak I_Na_ and its kinetics in hiPSC-CMs with *SCN10A* or *SCN5A* variant, but not in cells with the *CACNB2* variant, or WT and isogenic control cells. These findings indicate that cells with *SCN10A* and *SCN5A* variants were more susceptible to fever. Although mechanisms underlying the differential responses of the three BrS cell lines to hyperthermia remain unclear, these results may provide a theoretical basis for the future genotype-guided management for BrS patients with fever.

PKA, activated by cAMP, has been found to be the regulator of Nav1.5 expression through PKA and PKC signaling [32, 33]. Fever induced by LPS was further aggravated by PKA/PKC antagonists through the inhibition of p-TRPV1, linking PKA signaling to thermoregulation [34]. In our results, the treatment of PKA activator cAMP rescued the fever induced effects in arrhythmogeneity and peak I_Na_ in BrS cells with *SCN10A* and *SCN5A* variants, whereas the PKA antagonist H-89 exacerbated these effects. In addition, PKA expression decreased in BrS cells but increased in controls after fever treatment. These findings suggest that fever may worsen the BrS phenotype through PKA signaling, providing new mechanistic insight.

It should be noted that fever is not merely an elevation of body temperature but also involves complex inflammatory responses. In our study, the hyperthermia protocol (40 °C for 24 h) was designed to mimic the direct thermal component of fever, and our findings suggest that elevated temperature itself can exacerbate sodium channel dysfunction and arrhythmogeneity in BrS-hiPSC-CMs. Nevertheless, fever is typically accompanied by the release of pro-inflammatory mediators such as interleukins and tumor necrosis factors, which have been shown to modulate cardiac ion channel function and arrhythmia susceptibility [7, 35]. These cytokines or other fever-released factors may therefore act synergistically with hyperthermia to further aggravate the BrS phenotype. Future studies are warranted to dissect the relative contributions of direct thermal stress versus fever-associated mediators, as well as their potential interaction, in mediating fever-induced arrhythmic risk in BrS patients.

Since fever is a well-known hallmark of infection disease, fever-induced BrS may also be associated with inflammation. Recent studies indicated the possible relationship between BrS and inflammation. The C-reactive protein (CRP) levels in blood samples from symptomatic BrS patients were significantly higher than that in asymptomatic group [9]. Another study showed that BrS patients with acute cardiac inflammation frequently developed ventricular fibrillation episodes [10]. As the endotoxin of Gram-negative bacterial, LPS is commonly utilized to simulate infection and induce inflammation in studies. Our recent study demonstrated that LPS suppresses peak I_Na_ in hiPSC-CMs carrying *SCN10A* variants through ROS/PKC signaling [16]. In the present study, we applied LPS again to simulate inflammatory responses in hiPSC-CMs harboring *SCN10A*, *CACNB2*, or *SCN5A* variants. The present results showed that LPS exacerbated BrS phenotypes in hiPSC-CMs, including reduction of I_Na_ and I_Ca−L_ in cardiomyocytes with *SCN10A* and SCN5A variants or *CACNB2* variant, respectively. Of note, LPS showed no significant effect on I_Na_ in *CACNB2*-hiPSC-CMs and both I_Na_ and I_Ca−L_ in WT and isogenic cell lines, suggesting the sodium channel variants and calcium channel variant rendered sodium and calcium channels, respectively, sensitive to LPS. Reactive oxygen species (ROS) are normal byproducts of oxygen metabolism but rise dramatically under environmental stress, such as inflammation, heat or ultraviolet light, leading to cellular damage. It has been reported that the increase in ROS caused the reduction of I_Na_ in HEK cells expressing Nav1.5, and the effect of ROS could be blocked by NAD^+^ [36]. Similarly, we detected that ROS blocker rescued the I_Na_ and I_Ca−L_ in BrS-hiPSC-CMs exposed to LPS, indicating that the effect of LPS on cardiomyocytes was mediated by ROS production. Interestingly, LPS treatment also exacerbated recovery of I_Na_ and interval variability in WT, and NAC could rescue the effect on recovery but not arrhythmogeneity. This may be understood as that the effect of LPS on interval variability is not mediated by ROS. LPS is involved in various biologic processes, including generation of inflammatory factors, NF-kB signaling, oxidants, autophagy, and so on. So, the effect of LPS on interval variability of calcium transit may be mediated by other signaling pathways rather than ROS.

Inflammation damages cells and activates immune response, leading to the release of pro-inflammatory mediators, such as IL-6 [37]. IL-6 could increase the risk of QTc prolongation and Torsade de Pointes (TdP) [38], and may promote arrhythmias through directly regulating cardiac ion channels [14, 39, 40]. In the present study, the high concentration of IL-6R inhibitor TCZ reversed LPS-induced arrhythmogeneity in BrS1- and BrS3-hiPSC-CMs but not in BrS2-hiPSC-CMs, suggesting that the role of IL-6 in BrS may be genotype-dependent. IL-6 can exert both pro- and anti-inflammatory effects, depending on the context and the specific receptors in cells. Specifically, IL-6 plays a pro-inflammatory role in the acute phase response to infection and injury, promoting inflammation by inducing the production of acute phase proteins and activating other immune cells [41]. It also plays an anti-inflammatory role via activating a negative feedback loop that suppresses inflammation, and promotes clearance of apoptotic cells and tissue repair [42, 43]. In the present study, we identified IL-6 as a contributor to LPS-induced arrhythmic events in BrS-hiPSC-CMs with sodium channel variants. Whether its effects are mediated through pro- or anti-inflammatory effects remains to be clarified in future studies.

ROS can activate the redox sensitive transcription factor nuclear factor κB (NF-κB) signaling pathway, a major inducer of IL-6 generation and release [44, 45]. For example, exposure to particulate matter could increase IL-6 through TLR4/NADPH oxidase/ROS/NF-κB signaling [46], and cathelicidin-related antimicrobial peptides enhanced IL-6 secretion through the same pathway [47]. Our results also detected that the expression and secretion of IL-6 were increased after LPS challenge, and ROS inhibitor reduced the IL-6 levels in BrS-hiPSC-CMs, suggesting the effect of ROS on hiPSC-CMs with the *SCN10A* or *SCN5A* variant was mediated by IL-6. Building on our prior work linking ROS/PKC signaling to LPS-induced peak I_Na_ reduction in *SCN10A*-hiPSC-CMs [21], we confirmed this mechanism with isogenic controls and identified IL-6 as another downstream factor contributing to ion channel dysfunction or arrhythmogenesis. In addition, we detected similar effects of LPS/ROS on I_Ca−L_ but not peak I_Na_ in *CACNB2*-hiPSC-CMs. Another surprising finding is that TCZ failed to show any effect on the arrhythmogeneity induced by LPS in *CACNB2*-hiPSC-CMs, suggesting that a differential signaling was involved. These data suggest that the effects of LPS signaling may be gene variant related although other co-factors may also be involved.

Phosphorylation is a common biochemical modification, typically achieved by kinases acting on specific sites on proteins. Recent studies displayed the significant effects of phosphorylation on functions of Nav1.5 channels [48]. To further explore mechanisms by which LPS and hyperthermia reduced I_Na_ and enhanced arrhythmia-like events, we checked the total phosphorylation level in cells challenged by hyperthermia and LPS. It is a pity that no consistent results were obtained. The results cannot help explain the loss-of-function of sodium channel because the total phosphorylation level in *SCN5A* cells was reduced, but in *SCN10A* cells increased, although in both *SCN5A*- and *SCN10A*-cells, the peak I_Na_ was reduced. This can be understood as (1) the reduction of peak I_Na_ is not caused by phosphorylation or (2) the phosphorylation of a protein that caused I_Na_ reduction is not detected by the whole cell phosphorylation because phosphorylation and dephosphorylation can happen in many proteins when cells are challenged by fever of LPS. Therefore, we further checked beta-receptor level challenged by hyperthermia. Interestingly, hyperthermia reduced the expression of beta1-adrenoceptor in both *SCN10A* and *SCN5A* cells. It may be possible that fever can reduce adrenoceptor signaling and in turn reduce I_Na_ and exacerbate BrS. Here a question is how fever reduces adrenoceptor expression, which needs to be clarified in future studies.

Although *SCN10A* and *CACNB* variants are rarely identified in BrS-patients, comparing their effects with those of *SCN5A* variants provides important insights. All three variants similarly reduced V_max_ and peak I_Na_, but responses to hyperthermia and LPS challenge were genotype-dependent. Hyperthermia induced greater inhibition of I_Na_ and V_max_ in BrS3 than in BrS1 cells, while LPS caused stronger I_Na_ suppression but milder V_max_ reduction in BrS3 cells than in BrS1 cells. In contrast, neither stimulus significantly affected BrS2 cells. These findings suggest that gene variants or genotypes shape the severity of BrS phenotypes, especially under environmental stressors. Our findings underscore the potential importance of genotype-specific management strategies in BrS patients. Variants in *SCN5A* and *SCN10A* rendered sodium channels particularly vulnerable to febrile stress, which reinforces clinical recommendations that carriers of these mutations should avoid febrile states and initiate prompt antipyretic treatment at the onset of fever. In contrast, the *CACNB2* variant did not show a strong response to hyperthermia but was highly susceptible to inflammation-induced phenotypes, indicating that anti-inflammatory interventions may be especially relevant in this subgroup. These results highlight the potential value of preventive and therapeutic approaches according to the disease genotype.

Considering that arrhythmias in BrS patients occur mainly at rest, which suggests that heart beating frequency may play a role, we examined AP changes caused by hyperthermia and LPS challenge at different frequencies. Indeed, some frequency-dependent effects were detected. For example, in BrS1 cells, Vmax was more reduced by LPS at 1 and 2 Hz than 3 Hz, while this effect was not observed in BrS2 cells. On the contrary, LPS-induced APD50 changes were observed at lower frequencies in BrS2 cells but not in BrS1 cells. Moreover, fever effects on Vmax in both BrS1 and BrS2 cells did not show frequency-dependence. These data suggest that the influence of some environmental factors on BrS with certain genotype may be heart rate-dependent.

## Conclusions

The present study demonstrated that BrS-hiPSC-CMs with *SCN10A* and *SCN5A* variant displayed higher sensitivity to fever and inflammation challenge; PKA signaling mediated the effect of fever and ROS/IL-6 mediated the effect of inflammation on the phenotype of BrS-hiPSC-CMs; BrS-hiPSC-CMs with *CACNB2* variant displayed higher sensitivity to inflammation challenge but not fever; the gene mutation may impact effects of fever and inflammatory factors on BrS-phenotype.

## Study limitations

A major limitation of this study is that only one donor per genotype was used. Although the inclusion of isogenic correction lines provides a stringent control for genetic background and helps attribute the observed phenotypes to the specific variants, the influence of inter-individual variability on the results cannot be completely excluded and multiple healthy donors/patients are needed to address this issue. The difference between hiPSC-CMs and native cardiomyocytes should be also considered while interpreting the data. Some factors that affect cellular electrophysiology of cardiomyocytes in vivo have not been considered. The non-specific effects of the inhibitor and activator cannot be completely excluded. While our findings support mutation-driven mechanisms, we cannot completely rule out that protein-specific stress responses contribute to the observed differences of hyperthermia-or-LPS-induced changes in hiPS-CMs with different gene variants. Future studies will be required to address this question more comprehensively.

The data from this study indicate that hyperthermia and LPS exacerbate BrS phenotypes in different BrS-hiPSC-CM cell lines. However, some of these variants may not contribute to the BrS phenotype alone because of their high variant frequencies in the general population. Whether these variants contribute to disease phenotypes with other genetic or non-genetic factors were not clarified in the present study and need to be investigated in future studies.

The whole cell phosphorylation reflects the phosphorylation state of many proteins together, but not the phosphorylation of single protein. Some mechanistic interpretations, such as phosphorylation changes or the potential IL-6 dependence in *CACNB2*-hiPSC-CMs, remain speculative, as we did not map phosphorylation sites or measure phosphorylation of the channel proteins or identify specific signaling intermediates. Future studies should directly examine phosphorylation sites and state in channel proteins and signaling intermediates to validate these mechanistic hypotheses. LPS enhanced the arrhythmogeneity in BrS2 cells, but neither ROS nor IL-6 contributed to the effect. Which signaling is involved in this effect needs still be to clarified in future.

## Supplementary Information

Below is the link to the electronic supplementary material.

Supplementary Material 1.

## Data Availability

All data are available in the main text or the supplementary materials.
