# Supplementary material for: Phenotypes in Brugada syndrome with different genotypes triggered by fever or inflammation using gene-edited iPSCs
Source: Stem Cell Res Ther. 2025 Dec 1;16:670. doi: 10.1186/s13287-025-04793-6 (PMC12670799; doi:10.1186/s13287-025-04793-6)
Supplement: Supplementary file 1 — Supplementary Material 1. [file 13287_2025_4793_MOESM1_ESM.doc]

**Supplementary material**

**Phenotypes in Brugada Syndrome with Different Genotypes Triggered by Fever or Inflammation Using Gene-Edited iPSCs**

Yingrui Li^1,3,5^*****, Lena Rose^1,5^*, Timo Prädel^1,5^*, Mandy Kleinsorge^4,6^, Xuehui Fan^1,2,5^, Zenghui Meng^1,5^, Chen Yan^1,5^, Rui Liu^1^, Xinhao Lei^1^, Binyi Zhao^1^, Guoqiang Yang^1^, Zhenxing Liao^1^, Hendrik Dinkel^1^, Alexandra Viktoria Busley^4,6^, Rujia Zhong^1^, Feng Zhang^1^, Qiang Xu^1,2^, Lasse Maywald^1,5*^, Assem Aweimer^7^, Mengying Huang^1^, Alexander Moscu-Gregor^8^, Nazha Hamdani^9^, Luca Schneider^9^, Yeweynwuha Zemedi^9^, Saltanat Zhazykbayeva^9^, Alyssa Hohn^1^, Zhen Yang^1^, Lin Qiao^1^, Andreas Mügge^7^, Lukas Cyganek^4,6^, Xiaobo Zhou^1,2,5#^, Ibrahim Akin^1,5┼^, and Ibrahim El-Battrawy^7,9┼^

^1^First Department of Medicine, Faculty of Medicine, University Medical Centre Mannheim (UMM), Heidelberg University, 68167 Mannheim, Germany

**^2^Key Laboratory of Medical Electrophysiology of Ministry of Education and Medical Electrophysiological Key Laboratory of Sichuan Province,** Institute of Cardiovascular Research, Southwest Medical University, Luzhou, 646000 Sichuan, China

^3^Department of Cardiology, The Second Affiliated Hospital of Chongqing Medical University, Chongqing, 400010, China

^4^Stem Cell Unit, Clinic for Cardiology and Pneumology, University Medical Center Göttingen, 37075 Göttingen, Germany

^5^DZHK (German Center for Cardiovascular Research), Partner Site, Heidelberg-Mannheim, 68167 Mannheim, Germany

^6^DZHK (German Center for Cardiovascular Research), Partner Site, Göttingen, 37075 Göttingen, Germany

^7^Department of Cardiology and Angiology, Bergmannsheil University Hospitals, Ruhr University of Bochum, 44789 Bochum, Germany

^8^Center for Human Genetics and Laboratory Medicine, Martinsried, Germany

^9^Institute of Physiology, Ruhr University, Bochum, Germany; Molecular and Experimental Cardiology, Ruhr University, Bochum, Germany

*Equally contributed

# Corresponding author.

┼El-Battrawy and Akin share senior authorship.

**Address for correspondence**:

Xiaobo Zhou, MD. First Department of Medicine, University Medical Centre Mannheim, Theodor-Kutzer-Ufer 1-3, 68167 Mannheim, Germany. Phone: 0049-621-383-1448. Fax: 0049-621-383-1474. E-mail: xiaobo.zhou@medma.uni-heidelberg.de

Disclosures: none

**Materials and Methods**

**Ethics** Primary somatic cells were derived from skin biopsies or blood samples after written informed consent had been obtained. The study was approved by the Ethik-Kommission II der Universität Heidelberg, Medizinische Fakultät Mannheim (Investigation of the pathophysiology of cardiac arrhythmias using cardiomyocytes, vascular lining cells, nerve cells, and muscle cells from human induced pluripotent stem cells (hiPSCs) in various cardiovascular diseases, 2024-573, July 9, 2024). The study was conducted based on the Declaration of Helsinki 1975 of the World Medical Association, as revised in 2013.

**Generation of human induced pluripotent stem cells** Human hiPSC lines from a healthy donor and from three BrS patients were used in this study. Wild type iPSC line UMGi130-A clone 5 (isWT11.5, here abbreviated as WT) was generated from peripheral mononuclear blood cells of a healthy male donor using the integration-free Sendai virus and described previously [18]. The hiPSC cell lines UMGi126-A clone 23 (isBrSd1.23, here abbreviated as BrS1), UMGi119-A clone 1 (isBrSb2.1, here abbreviated as BrS2) and UMGi127-A clone 1 (isBrSe1.1, here abbreviated as BrS3) were generated from skin fibroblasts of BrS patients with *SCN10A* variant (NM_006514.4: c.3803G>A/p.R1268Q), *CACNB2* variant (NM_000724.4: c.425C>T/p.S142F), and *SCN5A* variant (NM_000335.5: c.3148G>A/p.A1050T) respectively, using the integration-free Sendai virus and described previously [19,20]. The variants in *SCN10A*, *CACNB2* and *SCN5A* were corrected by using CRISPR/Cas9 technology. For genome editing, early passage iPSCs (<p20) were selected. 300 pmol Alt-R CRISPR-Cas9 crRNA and 300 pmol Alt-R CRISPR-Cas9 tracrRNA were pre-assembled with 122 pmol Alt-R Hifi SpCas9 Nuclease 3NLS (all IDT DNA Technologies) to form the ribonucleoprotein complex. The mixture was combined with a single-stranded oligonucleotide for homology-directed repair. For SCN10A, three silent SNPs were designed within the protospacer region to prevent repeated Cas9 cleavage after the desired edit was achieved. For Nucleofection was performed with 2×106 iPSCs using the 4D Amaxa Nucleofector system (Lonza; program CA-137) and the P3 Primary Cell 4D-Nucleofector X Kit (Lonza) according to manufacturer’s instructions. Following nucleofection, iPSCs were replated into a Matrigel-coated (growth factor reduced, BD Biosciences) 6-well plate containing StemFlex medium supplemented with 2 µM thiazovivin (Merck) and 100 U/ml penicillin and 100 µg/ml streptomycin (Thermo Fisher Scientific). After 3 days, transfected iPSCs were singularized using the CellenOne single cell dispenser (Cellenion/Scienion) in StemFlex medium on Matrigel-coated 96-well plates. Successful genome editing was identified by Sanger sequencing. CRISPR-corrected isogenic iPSC lines UMGi126-A-1 clone 53A12 (isBrSd1-corr.53, here abbreviated as isogenic 1), UMGi119-A-1 clone 6 (isBrSb2-corr.6, here abbreviated as isogenic 2) and UMGi127-A-1 clone 2G6 (isBrSe1-corr.2G6, here abbreviated as isogenic 3) underwent pluripotency characterization, as previously described [21]. The hiPSC lines were differentiated into ventricular cardiomyocytes via WNT signaling modulation and subsequent metabolic selection and cultured for at least 50 days, as previously described [22]. Human iPSCs and derived cardiomyocytes were cultured in feeder-free and serum-free culture conditions in a humidified incubator at 37°C and 5% CO_2_. Subsequently, Western blot, patch clamp, calcium imaging and enzyme-linked immunosorbent assay were performed.

**Cell culture system and differentiation of hiPSCs**

Culture plates and dishes were coated with Matrigel (Corning). Culture medium of hiPSCs was TeSR-E8 (Stemcell Technologies, #05990) and the medium of hiPSC-CMs was RPMI 1640 Glutamax with Penicillin/Streptomycin, sodium pyruvate, ascorbic acid and B27 (Life Technologies, #17504001). ROCK inhibitor (Stemgent, #130-103-922) was added in E8 only after splitting for 24 hours. Frozen aliquots of hiPSCs were thawed and transferred to 6-well plate after being split for three times. The hiPSCs were cultured in TeSR-E8 and differentiation was performed when cells reached 85-95% confluence. CHIR99021 (Stemgent, #130-103-926) was added at the first day and IWP-2 (Stemgent, #72122) was added at the third day of differentiation to induce hiPSCs to differentiate into hiPSC-CMs. Beating cell colonies can be observed at around 6 to 8 days. Selection medium which contained sodium lactate and RPMI medium without glucose and glutamine was used at around 13 to 15 days. After using selection medium for 4 days, basic culture medium was used. At 50 to 60 days, cardiomyocytes were split from 6-well plates by 0.05% Trypsin-EDTA and used for further experiments.

**Western blot**

The hiPSC-CMs were split from 6-well plate by ice-cold RIPA lysis buffer (Sigma, #r0278) with protease inhibitor (Sigma-Aldrich #P8340-1ML). BCA protein assay reagents (Thermo Fisher Scientific, #23227) were utilized for measuring protein concentration. 20 μg protein were separated by sodium dodecyl sulfate polyacrylamide gel electrophoresis (SDS-PAGE) and electro-transferred to polyvinylidene difluoride (PVDF) membranes by semi-dry electrophoretic transfer cell (Bio-Rad). Membranes were blocked with 5% nonfat dry milk powder dissolved in Tris-buffered saline with 0.1% Tween-20 (TBST) at room temperature for 1 hour. Then, membranes were incubated with primary antibodies at 4°C overnight. On the next day, membranes were incubated with peroxidase-conjugated secondary antibodies dissolved in TBST at room temperature for 1 hour. Immunoreactive bands on the membrane were detected by enhanced chemiluminescence (Thermo Fisher Scientific, #1859697 and #1859700) by autoradiography. PKA (abcam #ab75991), IL-6 (R&D Systems #MAB206), β1-Adrenergic receptor (Alomone Labs #AAR-023), total protein phosphorylation anti–phospho-serine/threonine antibody (ECM Biosciences LLC; PP2551) and GAPDH (HyTest #5G4) primary antibodies as well as GAR (Sigma Aldrich #A0545) and GAM (Sigma Aldrich #A3682) secondary antibodies were used.

**Patch Clamp**

The whole-cell patch-clamp recording techniques was used to measure the peak sodium current (peak I_Na_), L-type calcium channel currents (I_Ca-L_) and action potentials (APs) at room temperature or 37℃ or 40℃. The electrode used for patch-clamp was pulled from borosilicate glass capillary (MTW 150F; World Precision Instruments, Sarasota, FL) by DMZ-Universal Puller (Zeitz-Instrumente Vertriebs GmbH, Martinsried, Germany). The EPC-8 amplifier (HEKA Elektronik) connected with a 16-bit A/D interface to a Pentium IBM clone computer was used for recordings. The signals were low-pass filtered (1 kHz) before 5 kHz digitization. The resistance of patch-pipettes ranged from 1–2 MΩ for current measurement and 3-4 MΩ for AP measurement. The electrode offset potentials was adjusted to zero before the pipettes touched the surface of cells. We usually waited for 1 to 3 minutes before recordings started to minimize the influence of rundown of recorded currents on the results. The ISO-3 multitasking patch-clamp program (MFK M. Friedrich) was used for data acquisition and analysis.

The current was normalized to the membrane capacitance to obtain current densities in pA/pF. The current density at -40 mV represented the expected largest sodium current (peak I_Na_), and the current density at 10 mV was utilized to represent the expected largest calcium current (I_Ca-L_). The current-voltage (I-V) relationship curve was produced by plotting the current density relative to respective voltages. Measuring the activation kinetics, the membrane conductance (Gm) was calculated by the formula $G_{m}=\frac{I_{m}}{(E-E_{rev})}$ (Im = current; E = applied potential; E_rev_ = reversal potential). The activation curve was produced by plotting G_m_/G_max_ to the respective voltages. For analyzing the inactivation kinetics, the channel availability was examined with pre-pulses from -120 to 40 mV. The inactivation curve was obtained by the method described above. V_0.5_, which is the voltage at which the conductance was half-maximal, was calculated. For measuring the recovery time constant of channels, the recorded and normalized currents were fitted with a 1st order exponential function to obtain the time constant (tau) of channel recovery. The tau value means the elapsed time at which the 63% of channels can be activated again. APs were measured in the current-clamp mode. Different parameters of APs were measured, including resting potential (RP), AP amplitude (APA), the maximal velocity of depolarization (V_max_), the AP duration at 50% repolarization (APD50) and the AP duration at 90% repolarization (APD90)

The protocols for recording peak I_Na_ and I_Ca-L_ were displayed in **figure S1**. The bath solution for recording peak I_Na_, I_Ca-L_ and APs were described in detail in our recent studies [19,23].

**Calcium transient imaging**

To measure the intracellular Ca^2+^ transients, the fluorescent Ca^2+^-indicator Fluo-3 AM was utilized. 10 µM Fluo-3 was incubated with cells at room temperature for 10 minutes, which should be protected from light. Then, the cells were washed for 4-5 times and kept in PSS at room temperature for about 30 minutes for de-esterification before measurements. The fluorescence of the cells was measured by using a Cairn Optoscan calcium imaging system (Cairn Research, UK). Fluorescence was excited by 488 nm and emitted at 520 nm. Arrhythmic events are defined as the presence of early afterdepolarization (EAD)-like or delayed afterdepolarization (DAD)-like or triggered beat events.

**Enzyme-linked immunosorbent assay**

The concentration of IL-6 in hiPSC-CMs culture supernatants was measured by using the enzyme-linked immunosorbent assay (ELISA) with a human IL-6 ELISA Kit (RayBiotech). 100 µl of each standard and sample solution were added into 96 well plate. The wells were covered and incubated for 2.5 hours at room temperature with gentle shaking. Then, the solution was discarded and cells were washed 4 times. The prepared biotinylated antibody was added and incubated for 1 hour at room temperature with gentle shaking. After repeating wash steps, Streptavidin solution was added with gentle shaking for 45 minutes at room temperature. The wash step was repeated and TMB One-Step Substrate Reagent was added in the dark with gentle shaking for 30 minutes at room temperature. Then, 50 µl of Stop Solution was added. The signal was read at 450 nm immediately by microplate reader. The final concentration of IL-6 in samples was calculated with the standard concentrations.

**Immunofluorescence staining**

Intracellular oxidants generation was assessed using 2',7'-Dichlorofluorescin diacetate (DCFH-DA), a cell-permeable non-fluorescent probe that can be converted into the fluorescent compound 2',7'-dichlorofluorescein (DCF) by oxidants. Following a 24-hour incubation with LPS, the cells underwent two washes and were subsequently exposed to a 5 μM DCFH-DA solution in serum-free medium at 37°C for 30 minutes in a dark environment. The fluorescence of DCF in the cells within the dish was evaluated using a fluorescence microscope (BX51; Olympus Corp.).

**Drugs**

Lipopolysaccharides (LPS, 2μg/ml, 24 hours, #L2630), N-Acetyl-L-cysteine (NAC, 1mM, 24 hours, #A7250), H-89 (10μM, 24 hours, #B1427) were purchased from Sigma-Aldrich. 8-Bromo-cAMP (cAMP, 5μM, 24 hours, #B002-05) was purchased from Biolog. Tocilizumab (TCZ, 10-10000ng/ml, 24 hours, #A2012) was purchased from SelleckChem.

**Statistical analysis** All data, if not otherwise stated, are shown as mean ± SEM and were analyzed by SigmaPlot 14.0 (Systat GmbH, Germany). By analyzing the data with the Normality test, it was decided whether parametric or non-parametric tests were used for analysis. Unpaired student's t-test analysis was conducted to compare two independent groups with normal distribution. One-way analysis of variance (ANOVA) followed with Holm-Sidak post-test for multiple comparisons was used for comparing more than two groups. The Fisher-test was applied for comparing categorical variables. p values<0.05 were considered statistically significant.

**Supplementary figures and tables**


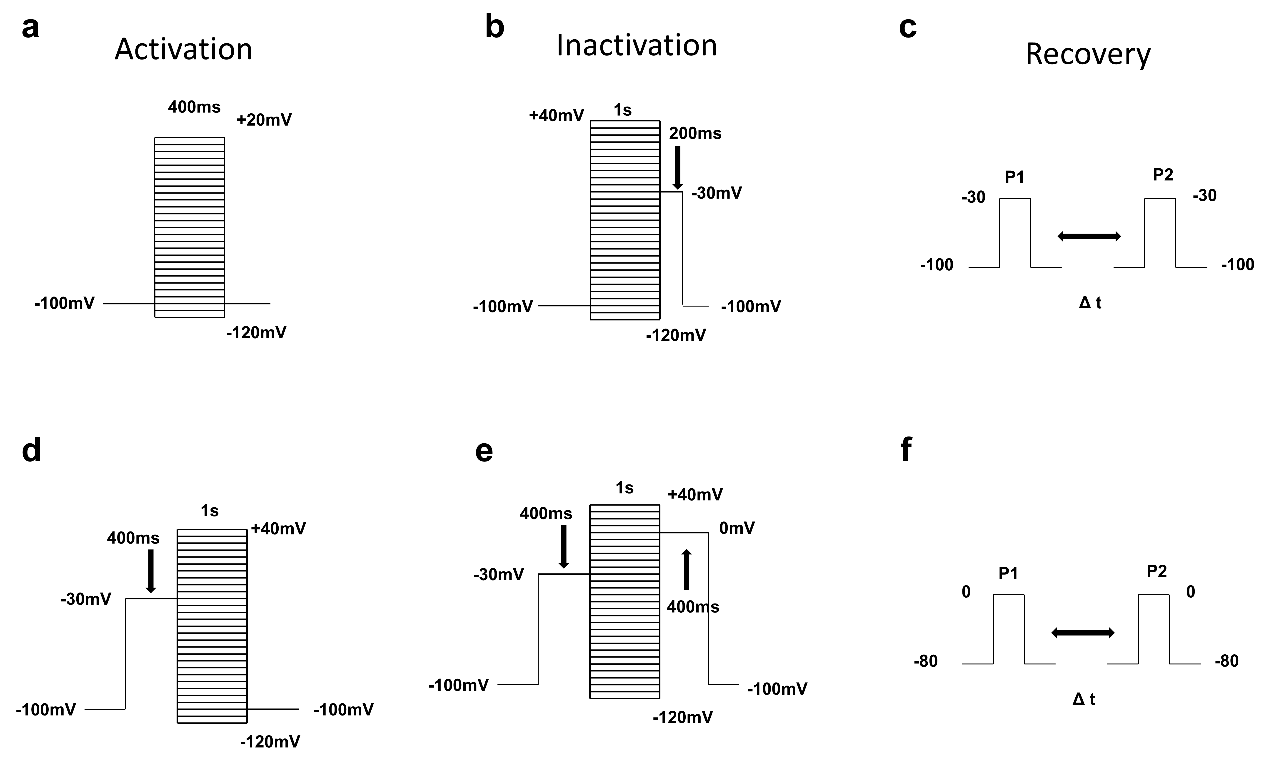


Fig. S1.

**Protocols for measuring peak sodium channel currents (I_Na_) and L-type calcium channel currents (I_Ca-L_).** (a) Protocol for measuring peak current and activation of I_Na_. (b) Protocol for measuring inactivation of I_Na_. (c) Protocol for measuring recovery of I_Na_. (d) Protocol for measuring peak current and activation of I_Ca-L_. (e) Protocol for measuring inactivation of I_Ca-L_. (f) Protocol for measuring recovery of I_Ca-L_.


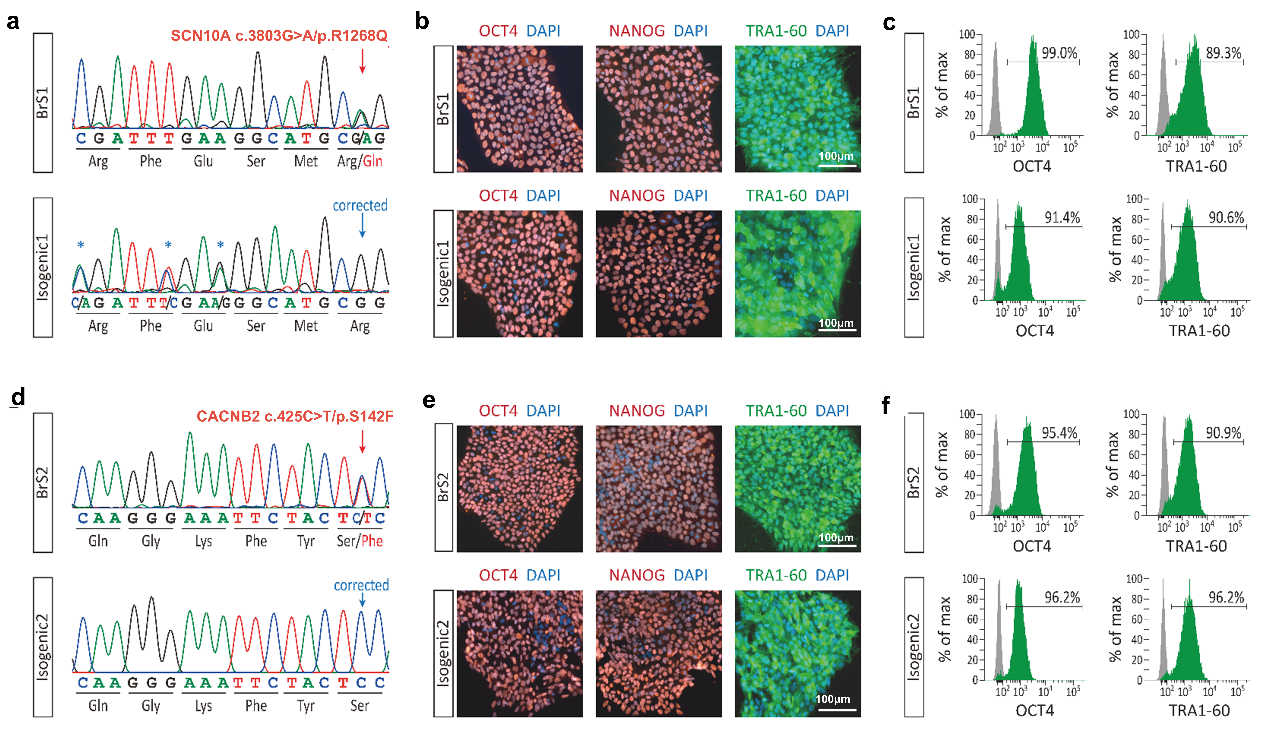


Fig. S2.

**Sanger sequencing of patient-derived cells and expression of pluripotency markers.** (a) Sanger sequencing of patient-derived cells confirmed the presence of the *SCN10A* variant and its correction in CRISPR-edited isogenic controls. (b) Immunofluorescence analysis of pluripotency markers in the BrS1 and Isogenic1 hiPSCs. (c) Flow cytometry for pluripotency markers in hiPSCs from BrS1 and Isogenic1 cells. (d) Sanger sequencing of patient-derived cells confirmed the presence of the *CACNB2* variant and its correction in CRISPR-edited isogenic controls. (e) Immunofluorescence analysis of pluripotency markers in the BrS2 and Isogenic2 hiPSCs. (f) Flow cytometry for pluripotency markers in hiPSCs from BrS2 and Isogenic2 cells.Type or paste caption here. Create a page break and paste in the Figure above the caption.


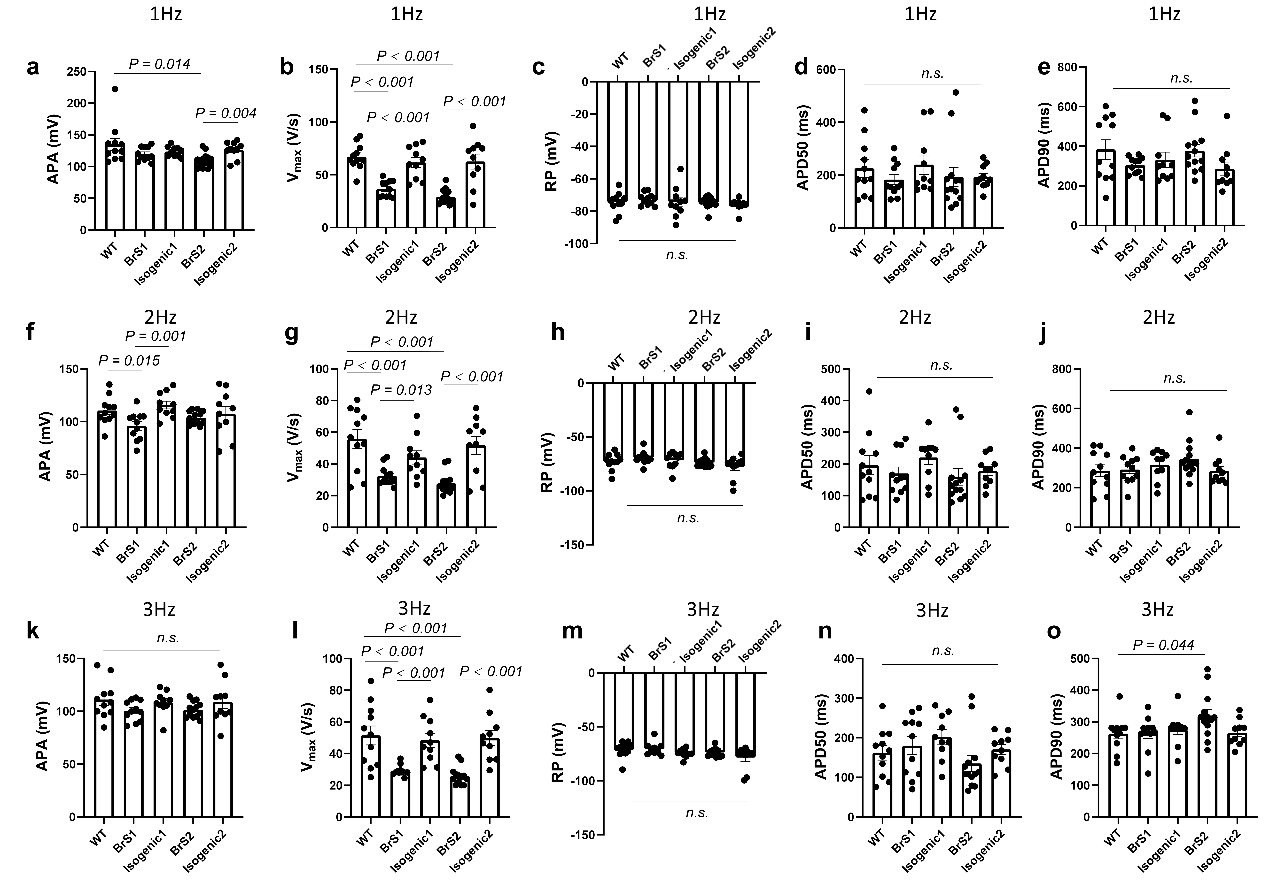


Fig. S3.

**Changes of action potential in hiPSC-CMs from the BrS patients.** Action potentials were measured at room temperature in 1 Hz (a-e), 2 Hz (f-j) and 3 Hz (k-o) in hiPSC-CMs from a healthy donor (WT), a BrS-patient with the variant in *SCN10A* (BrS1), a BrS-patient with the variant in *CACNB2* (BrS2) and the variant-corrected cell lines (Isogenic1 and Isogenic2). (a) Mean values of the amplitude (APA) of APs at 1 Hz in each group. (b) Mean values of the maximal depolarization velocity (V_max_) of APs at 1 Hz in each group. (c) Mean values of the resting potential (RP) of APs at 1 Hz in each group. (d) Mean values of repolarization at 50% (APD50) of APs at 1 Hz in each group. (e) Mean values of repolarization at 90% (APD90) of APs at 1 Hz in each group. (f) Mean values of the APA of APs at 2 Hz in each group. (g) Mean values of the V_max_ of APs at 2 Hz in each group. (h) Mean values of the RP of APs at 2 Hz in each group. (i) Mean values of APD50 of APs at 2 Hz in each group. (j) Mean values of APD90 of APs at 2 Hz in each group. (k) Mean values of the APA of APs at 3 Hz in each group. (l) Mean values of the V_max_ of APs at 3 Hz in each group. (m) Mean values of the RP of APs at 3 Hz in each group. (n) Mean values of APD50 of APs at 3 Hz in each group. (o) Mean values of APD90 of APs at 3 Hz in each group. The p values are determined by One way-ANOVA with Holm-Sidak post-test.


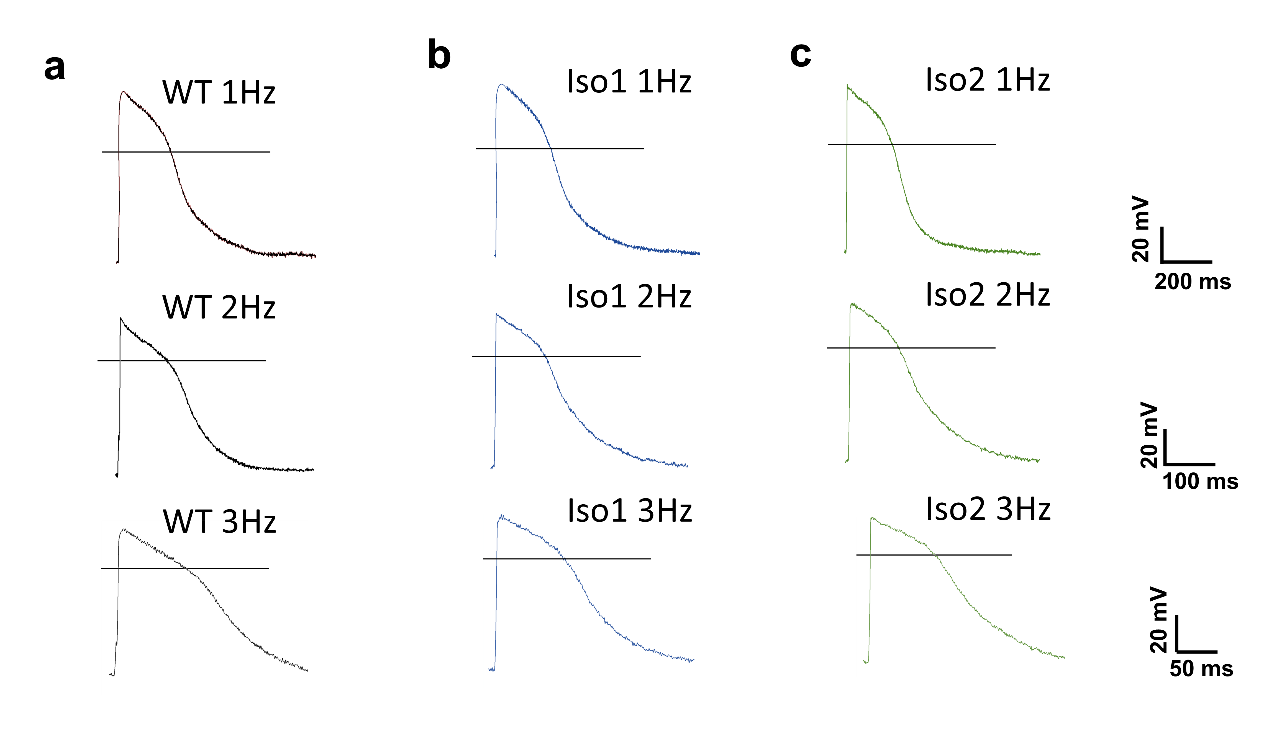


Fig. S4.

**Representative traces of action potentials of WT and isogenic cell lines.** Action potentials were measured at room temperature in 1 Hz, 2 Hz and 3 Hz in hiPSC-CMs from a healthy donor (WT) and the variant-corrected cell lines (Iso1 and Iso2).


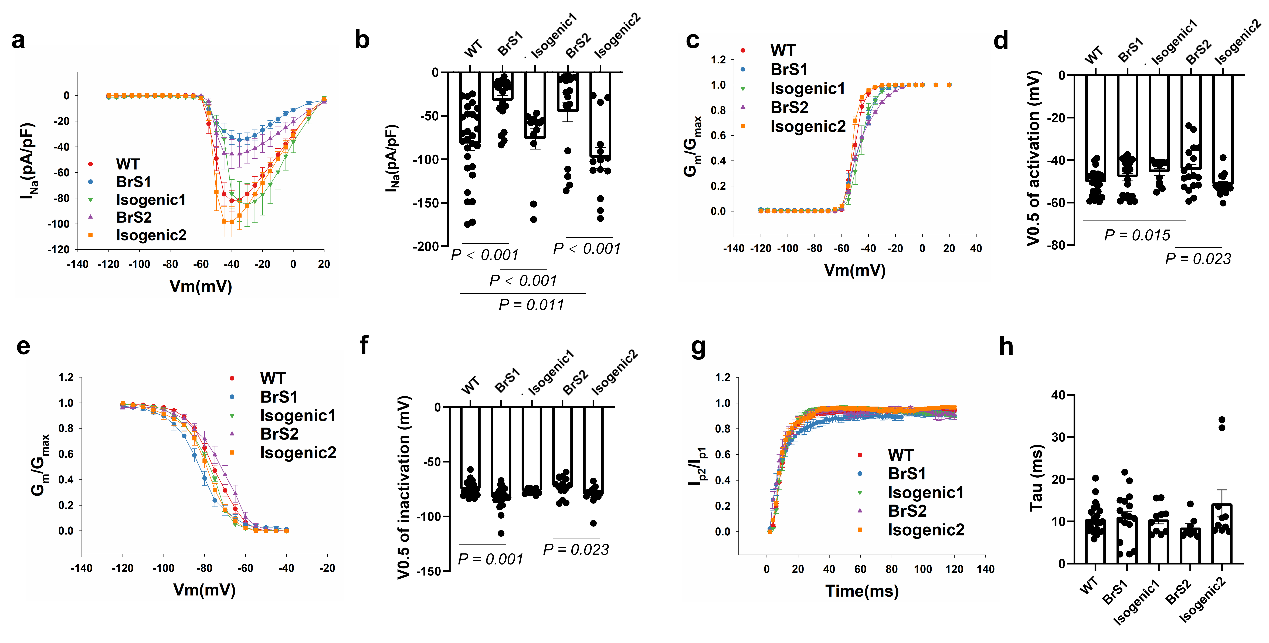


Fig. S5.

**The hiPSC-CMs from BrS patients displayed loss-of-function of sodium channels.** Peak sodium channel currents (I_Na_) were measured at room temperature in hiPSC-CMs from a healthy donor (WT), a BrS-patient with the variant in *SCN10A* (BrS1), a BrS-patient with the variant in *CACNB2* (BrS2) and the variant-corrected cell lines (Isogenic1 and Isogenic2). (a) Current-voltage (I-V) relationship curves of peak I_Na_ in each group. (b) Mean values of peak I_Na_ at -40 mV in each group. (c) Activation curves of peak I_Na_ in each group. (d) Mean values of potential at 50% activation (V0.5) in each group. (e) Inactivation curves of peak I_Na_ in each group. (f) Mean values of potential at 50% inactivation (V0.5) in each group. (g) Recovery curves of peak I_Na_ in each group. (h) Mean values of time constant (Tau) of recovery from inactivation in each group. The p values are determined by One way-ANOVA with Holm-Sidak post-test.


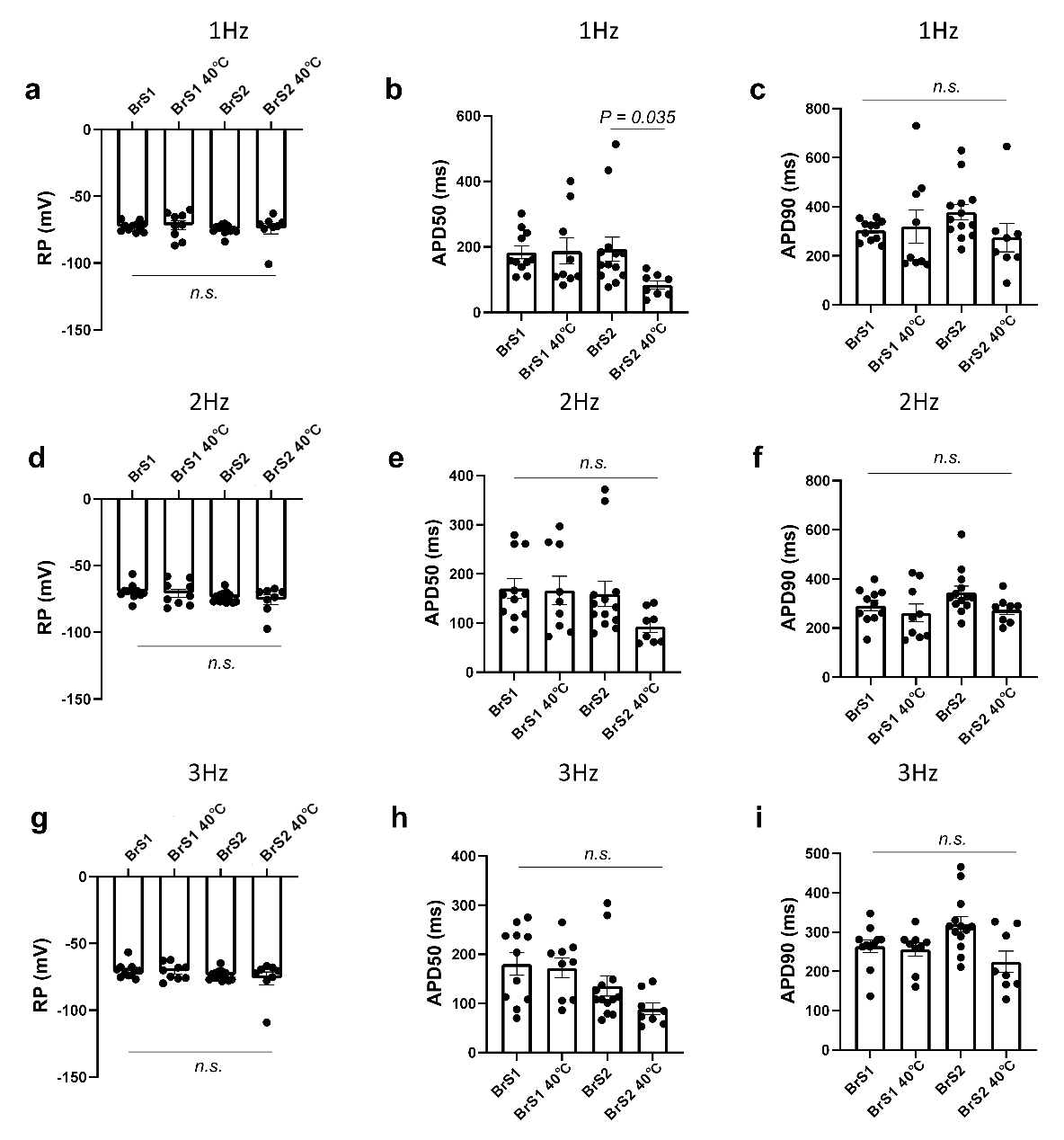


Fig. S6.

**Changes of action potential in BrS cell lines with hyperthermia treatment.** Action potentials were measured in hiPSC-CMs from the BrS-patients at 37℃ (BrS1 and BrS2) and 40℃ for 24 h (BrS1 40℃ and BrS2 40℃) at 1 Hz (a-c), 2 Hz (d-f) and 3 Hz (g-i). (a) Mean values of the resting potential (RP) in 1 Hz in each group. (b) Mean values of repolarization at 50% (APD50) of APs in 1 Hz in each group. (c) Mean values of repolarization at 90% (APD90) of APs in 1 Hz in each group. (d) Mean values of the RP in 2 Hz in each group. (e) Mean values of APD50 of APs in 2 Hz in each group. (f) Mean values of APD90 of APs in 2 Hz in each group. (g) Mean values of the RP in 3 Hz in each group. (h) Mean values of APD50 of APs in 3 Hz in each group. (i) Mean values of APD90 of APs in 3 Hz in each group. The p values are determined by One way-ANOVA with Holm-Sidak post-test.


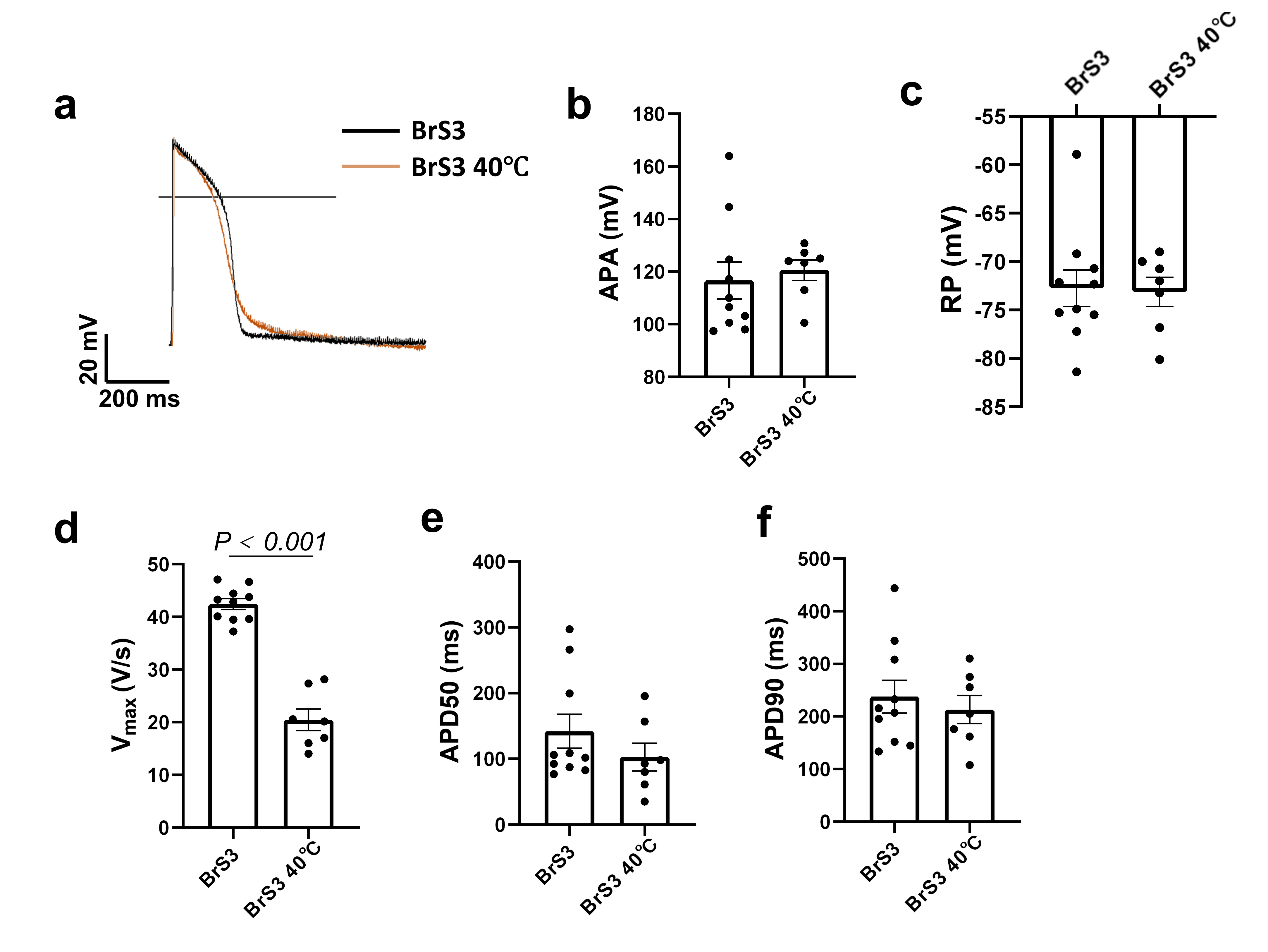


Fig. S7.

**The effect of hyperthermia and LPS on action potentials (APs) of BrS3.** The APs was measured in hiPSC-CMs from the a BrS-patient (BrS3) at 37℃ and 40℃ for 24 h. (a) Representative traces and parameters of APs in each group. (b) Mean values of the amplitude (APA) of APs in each group. (c) Mean values of the resting potential (RP) in 1 Hz in each group. (d) Mean values of maximal depolarization velocity (V_max_) of APs in each group. (e) Mean values of repolarization at 50% (APD50) of APs in 1 Hz in each group. (f) Mean values of repolarization at 90% (APD90) of APs in 1 Hz in each group. The p values are determined by unpaired Student’s t-test.


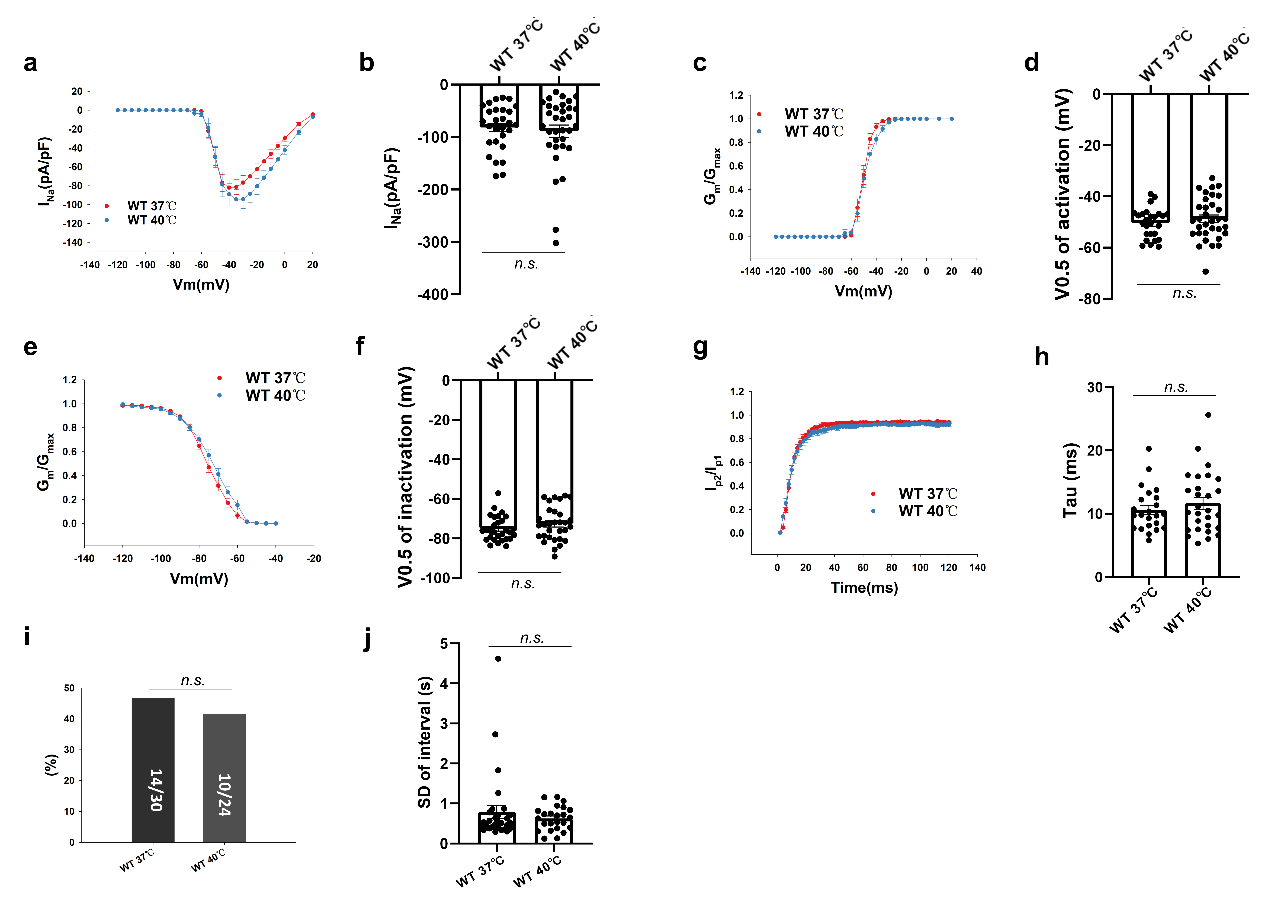


Fig. S8.

**The effect of hyperthermia on peak sodium channel currents (I_Na_) of WT.** I_Na_ was measured at 37℃ and 40℃ in WT cell line. (a) Current-voltage (I-V) relationship curves of peak I_Na_ in each group. (b) Mean values of peak I_Na_ at -40 mV in each group. (c) Activation curves of peak I_Na_ in each group. (d) Mean values of potential at 50% activation (V0.5) in each group. (e) Inactivation curves of peak I_Na_ in each group. (f) Mean values of potential at 50% inactivation (V0.5) in each group. (g) Recovery curves of peak I_Na_ in each group. (h) Mean values of time constant (Tau) of recovery from inactivation in each group. (i) The percentage of cells showing arrhythmic events (EAD-like events or triggered events) in each group. (j) Mean values of interval variability in each group. The p values are determined by the unpaired Student’s t-test.


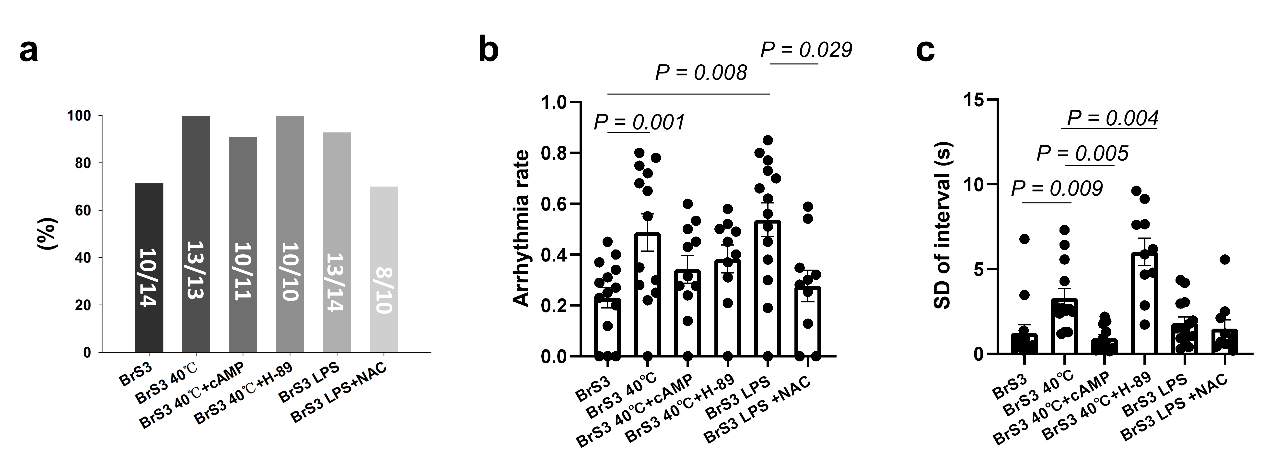


Fig. S9.

**The effect of hyperthermia or LPS on arrhythmogeneity of BrS3 cells.** The arrhythmogeneity was measured by calcium transients in hiPSC-CMs from the a BrS-patient (BrS3) at 37℃ and 40℃ for 24 h and 40℃ with PKA inhibitor (H-89, 10 µM, 24 hours) or activator (8-Bromo-cAMP, 5 µM, 24 hours), or in presence of LPS (2 μg/ml, 24 hours) and LPS plus oxidants blocker (NAC, 1 mM, 24 hours). (a) The percentage of cells showing arrhythmic events (EAD-like events or triggered events) in each group of BrS3. (b) The arrhythmia event rate in 100 seconds in each group of BrS3. (c) Mean values of interval variability in each group of BrS3. The p values are determined by One way-ANOVA with Holm-Sidak post-test (b, c) or Fischer-test (a).





Fig. S10.

**Representative traces of spontaneous calcium transients of WT, BrS and isogenic cell lines.** Spontaneous calcium transients was measured with or without hyperthermia, cAMP, H-89, LPS, NAC or TCZ treatment in hiPSC-CMs from a healthy donor (WT), a BrS-patient with the variant in *SCN10A* (BrS1), a BrS-patient with the variant in *CACNB2* (BrS2), a BrS-patient with the variant in *SCN5A* (BrS3) and the variant-corrected cell lines (Iso1 and Iso2). (a1-a6) Representative traces of spontaneous calcium transients of WT. (b1-b16) Representative traces of spontaneous calcium transients of BrS1. (c1-c6) Representative traces of spontaneous calcium transients of Iso1. (d1-d9) Representative traces of spontaneous calcium transients of BrS2. (e1-e4) Representative traces of spontaneous calcium transients of Iso2. (f1-f10) Representative traces of spontaneous calcium transients of BrS3.


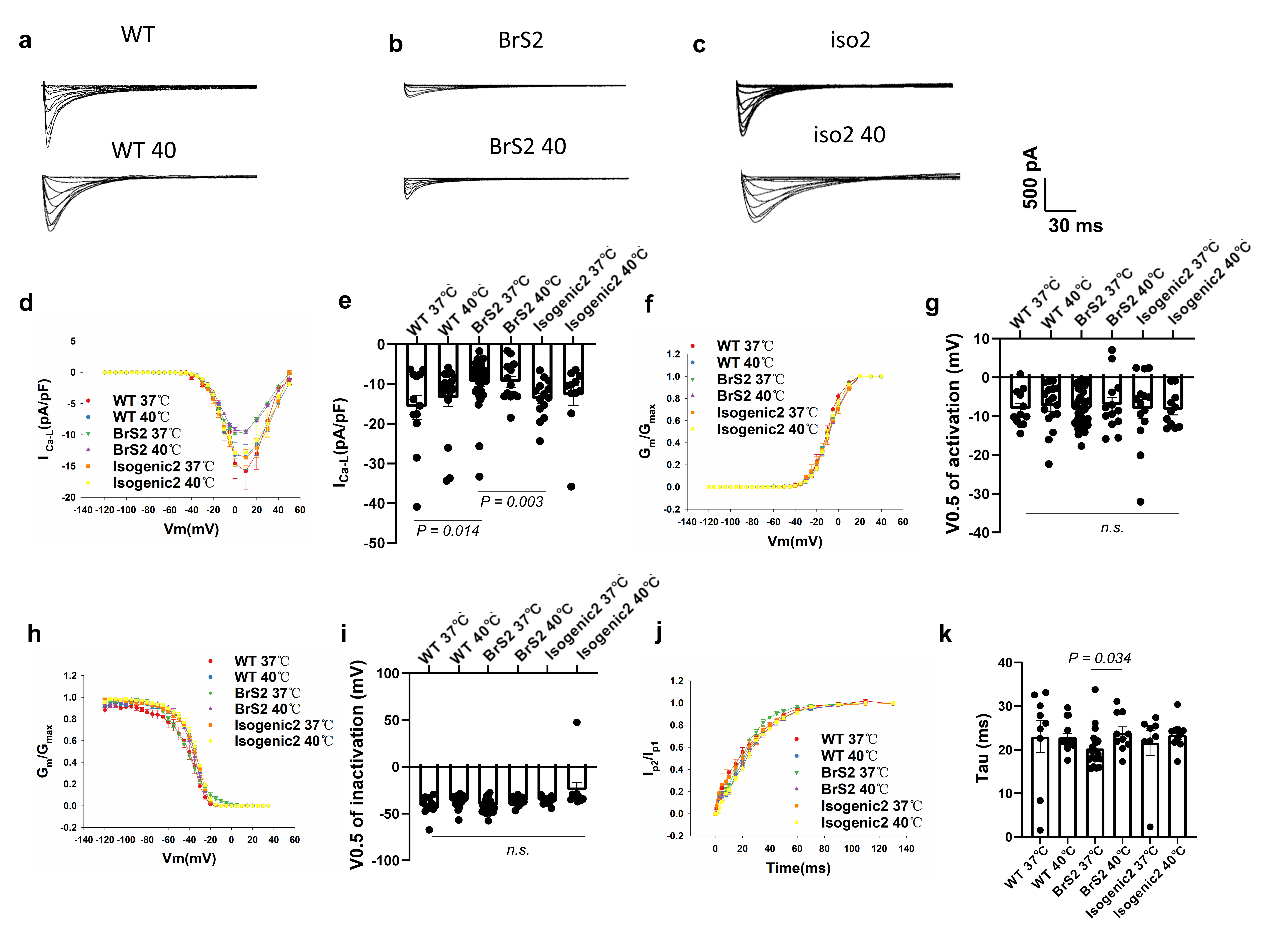


Fig. S11.

**The effect of hyperthermia on L-type calcium channel currents (I_Ca-L_) of WT and isogenic cell lines.** I_Ca-L_ was measured at 37℃ and 40℃ in WT and isogenic cell lines. (a-c) Representative traces of L-type calcium channel currents (I_Ca-L_) of each group. (d) Current-voltage (I-V) relationship curves of I_Ca-L_ in each group. (e) Mean values of I_Ca-L_ at 10 mV in each group. (f) Activation curves of I_Ca-L_ in each group. (g) Mean values of potential at 50% activation (V0.5) in each group. (h) Inactivation curves of I_Ca-L_ in each group. (i) Mean values of potential at 50% inactivation (V0.5) in each group. (j) Recovery curves of I_Ca-L_ in each group. (k) Mean values of time constant (Tau) of recovery from inactivation in each group. The p values are determined by One way-ANOVA with Holm-Sidak post-test.


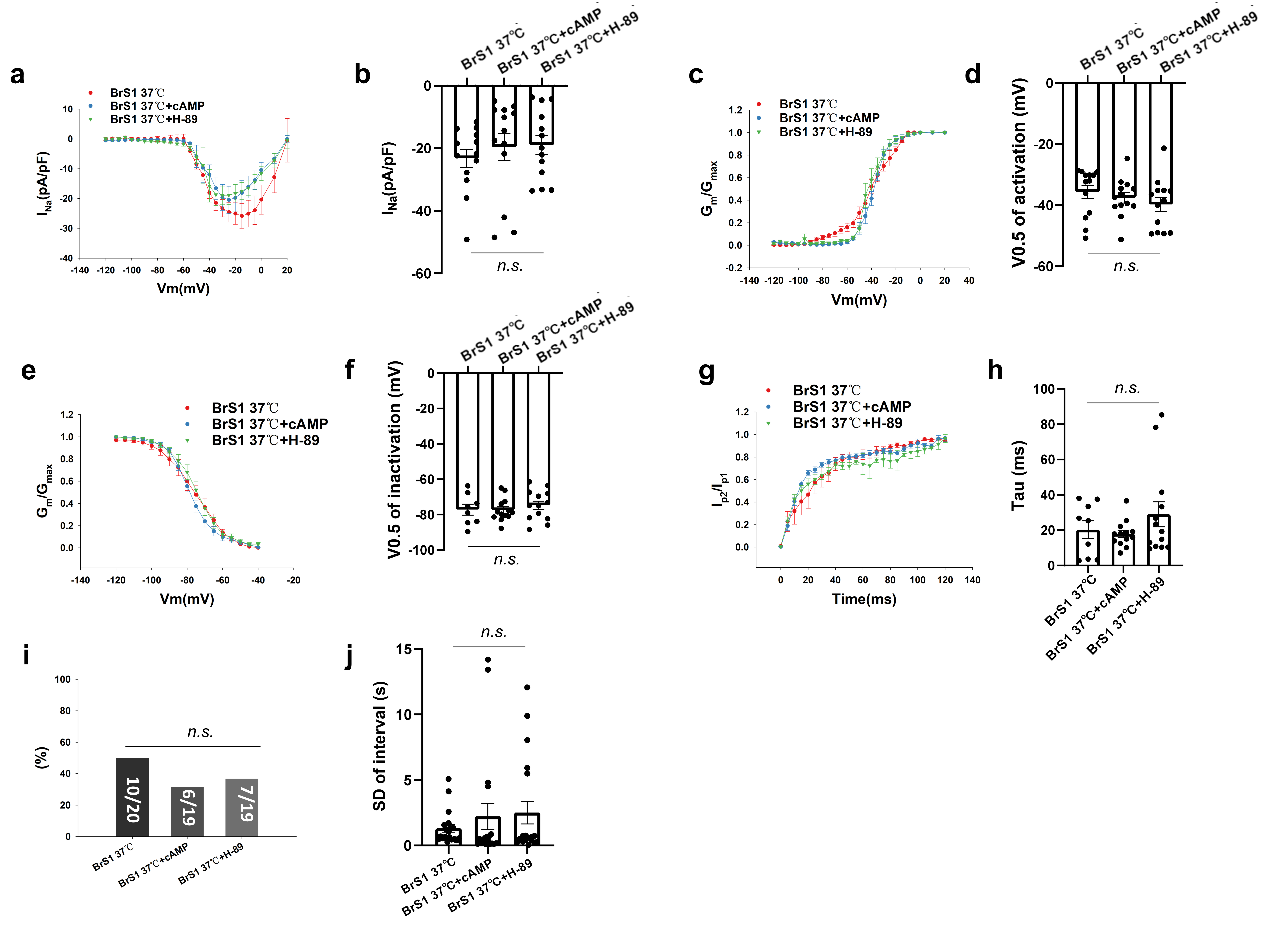


Fig. S12.

**The effect of PKA inhibitor (H-89) or activator (8-Bromo-cAMP) on peak sodium channel currents (I_Na_) of BrS1.** I_Na_ was measured at 37℃ in BrS1 cell line with PKA inhibitor (H-89, 10 µM, 24 hours) or activator (8-Bromo-cAMP, 5 µM, 24 hours). (a) Current-voltage (I-V) relationship curves of peak I_Na_ in each group. (b) Mean values of peak I_Na_ at -40 mV in each group. (c) Activation curves of peak I_Na_ in each group. (d) Mean values of potential at 50% activation (V0.5) in each group. (e) Inactivation curves of peak I_Na_ in each group. (f) Mean values of potential at 50% inactivation (V0.5) in each group. (g) Recovery curves of peak I_Na_ in each group. (h) Mean values of time constant (Tau) of recovery from inactivation in each group. (i) The percentage of cells showing arrhythmic events (EAD-like events or triggered events) in each group. (j) The mean values of interval variability in each group. The p values are determined by One way-ANOVA with Holm-Sidak post-test (b, d, f, h, j) or Fischer-test (i).


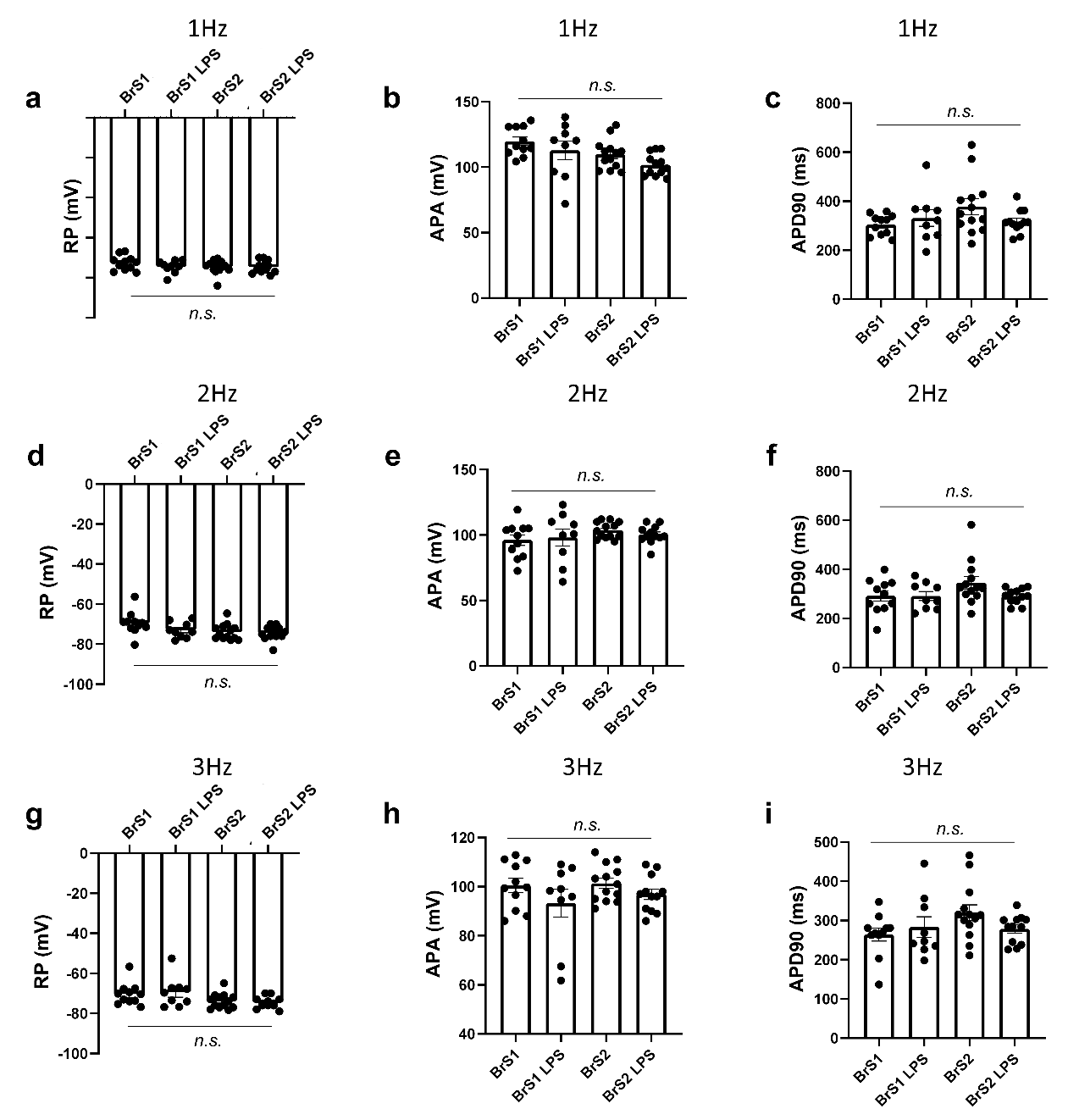


Fig. S13.

**Changes of action potential in BrS hiPSC-CMs treated with LPS.** Action potentials were measured in hiPSC-CMs from the BrS-patients at 37℃ (BrS1 and BrS2) and 37℃ with LPS treatment (2 μg/ml, 24 hours) (BrS1 LPS and BrS2 LPS) at 1 Hz (a-c), 2 Hz (d-f) and 3 Hz (g-i). (a) Mean values of the resting potential (RP) in 1 Hz in each group. (b) Mean values of amplitude (APA) of APs in 1 Hz in each group. (c) Mean values of repolarization at 90% (APD90) of APs in 1 Hz in each group. (d) Mean values of the RP in 2 Hz in each group. (e) Mean values of APA of APs in 2 Hz in each group. (f) Mean values of APD90 of APs in 2 Hz in each group. (g) Mean values of the RP in 3 Hz in each group. (h) Mean values of APA of APs in 3 Hz in each group. (i) Mean values of APD90 of APs in 3 Hz in each group. The p values are determined by One way-ANOVA with Holm-Sidak post-test.


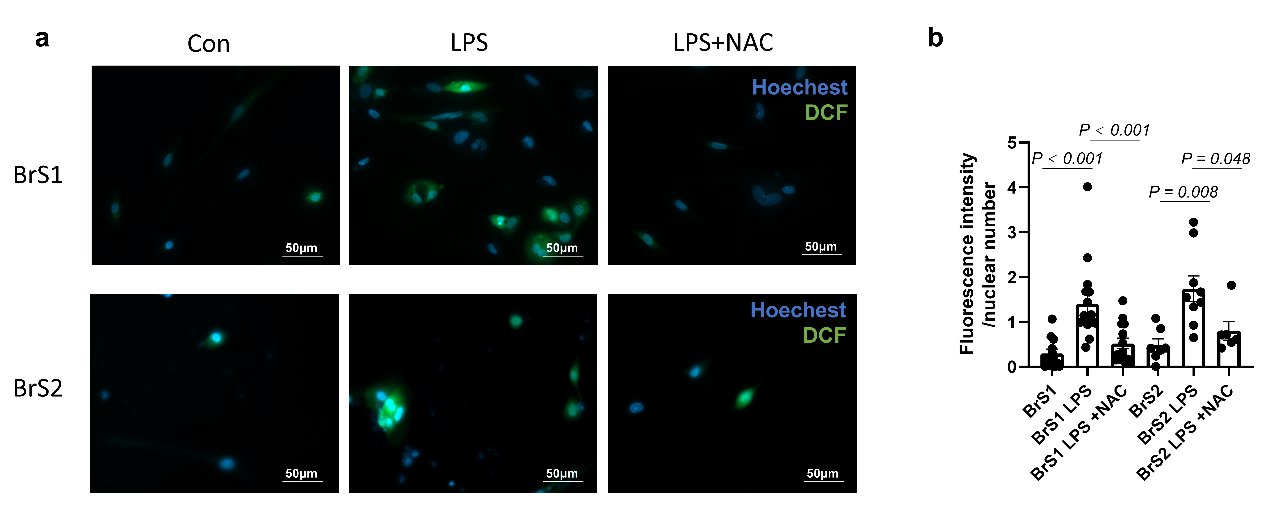


Fig. S14.

**LPS induced oxidants generation in BrS1-and BrS2.** The 2’,7’-dichlorofluorescein (DCF) was measured in BrS1 and BrS2 cell lines in presence of LPS (2 μg/ml, 24 hours) and LPS plus ROS blocker (NAC, 1 mM, 24 hours). (a) Representative images of fluorescence intensity in each group. (b) The ratio of fluorescence intensity and nuclear number in each group. The p values are determined by One way-ANOVA with Holm-Sidak post-test. Scar bar = 50 μm.


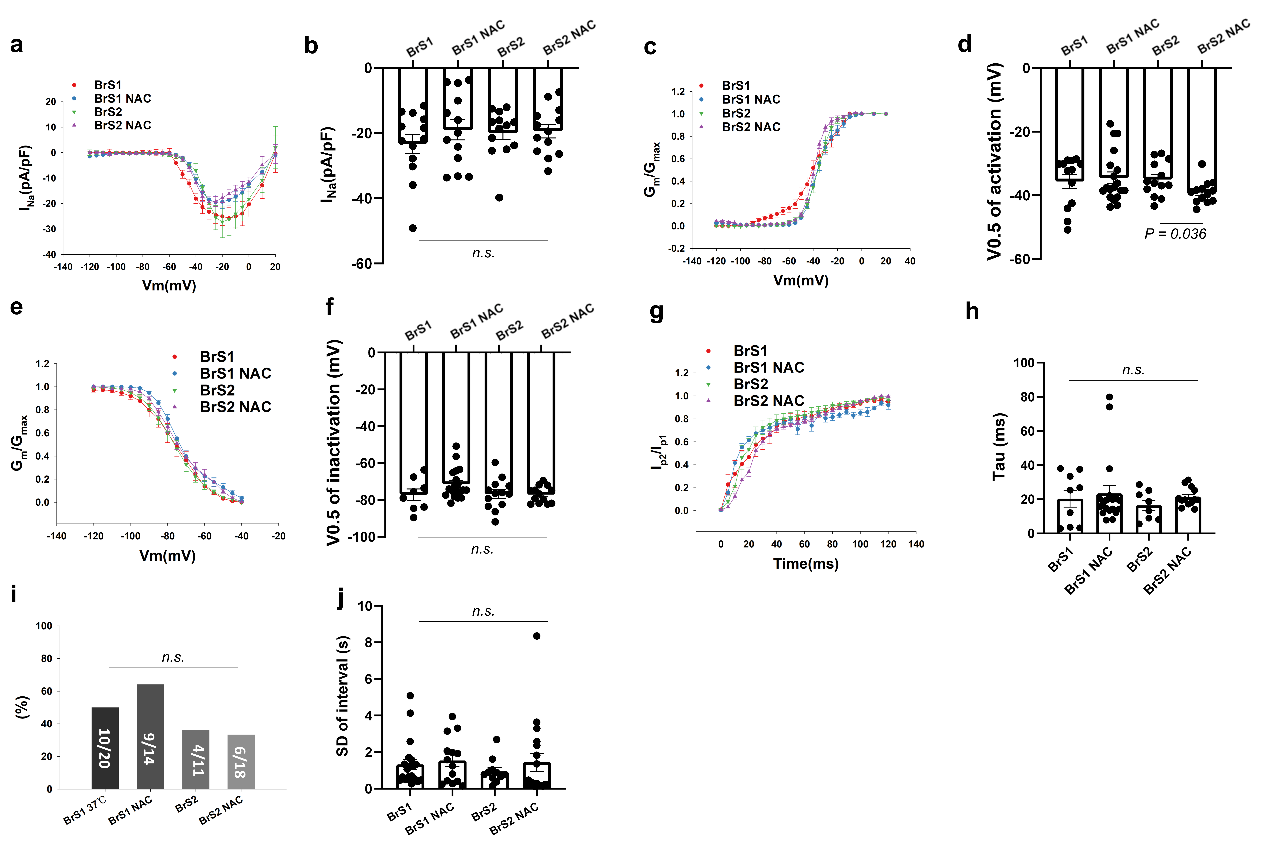


Fig. S15.

**The effect of NAC on peak sodium channel currents (I_Na_) of BrS1 and BrS2.** I_Na_ was measured with oxidants blocker (NAC, 1 mM, 24 hours) in BrS1 and BrS2 cell lines. (a) Current-voltage (I-V) relationship curves of peak I_Na_ in each group. (b) Mean values of peak I_Na_ at -40 mV in each group. (c) Activation curves of peak I_Na_ in each group. (d) Mean values of potential at 50% activation (V0.5) in each group. (e) Inactivation curves of peak I_Na_ in each group. (f) Mean values of potential at 50% inactivation (V0.5) in each group. (g) Recovery curves of peak I_Na_ in each group. (h) Mean values of time constant (Tau) of recovery from inactivation in each group. (i) The percentage of cells showing arrhythmic events (EAD-like events or triggered events) in each group. (j) Mean values of interval variability in each group. The p values are determined by One way-ANOVA with Holm-Sidak post-test (b, d, f, h, k) or Fischer-test (j).


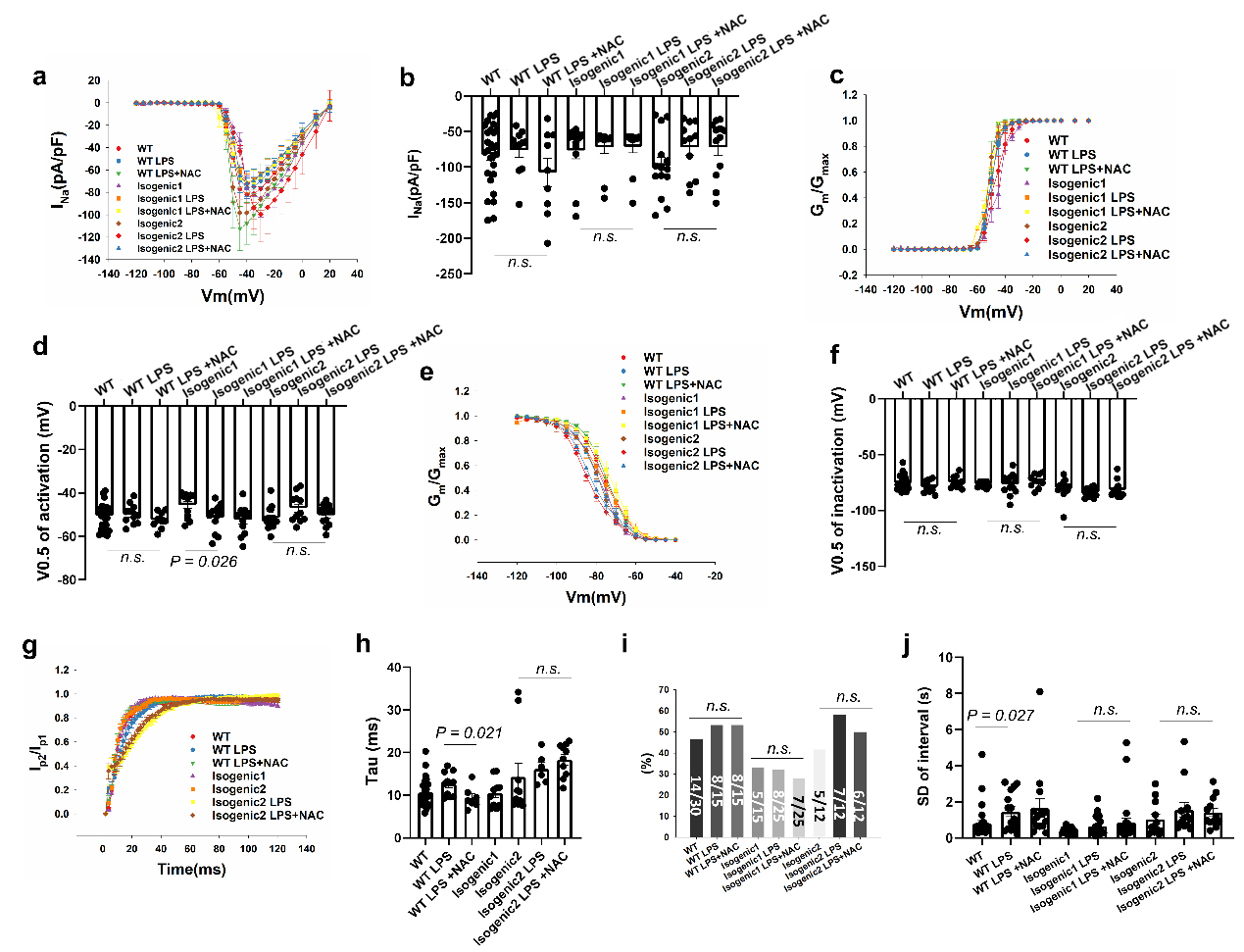


Fig. S16.

**The effect of LPS on peak sodium channel currents (I_Na_) of WT and isogenic cell lines.** I_Na_ was measured in WT and isogenic cells with LPS (2 μg/ml, 24 hours) and ROS blocker (NAC, 1 mM, 24 hours) treatment. (a) Current-voltage (I-V) relationship curves of peak I_Na_ in each group. (b) Mean values of peak I_Na_ at -40 mV in each group. (c) Activation curves of peak I_Na_ in each group. (d) Mean values of potential at 50% activation (V0.5) in each group. (e) Inactivation curves of peak I_Na_ in each group. (f) Mean values of potential at 50% inactivation (V0.5) in each group. (g) Recovery curves of peak I_Na_ in each group. (h) Mean values of time constant (Tau) of recovery from inactivation in each group. (i) The percentage of cells showing arrhythmic events (EAD-like events or triggered events) in each group. (j) Mean values of interval variability in each group. The p values are determined by One way-ANOVA with Holm-Sidak post-test (b, d, f, h, j) or Fischer-test (i).


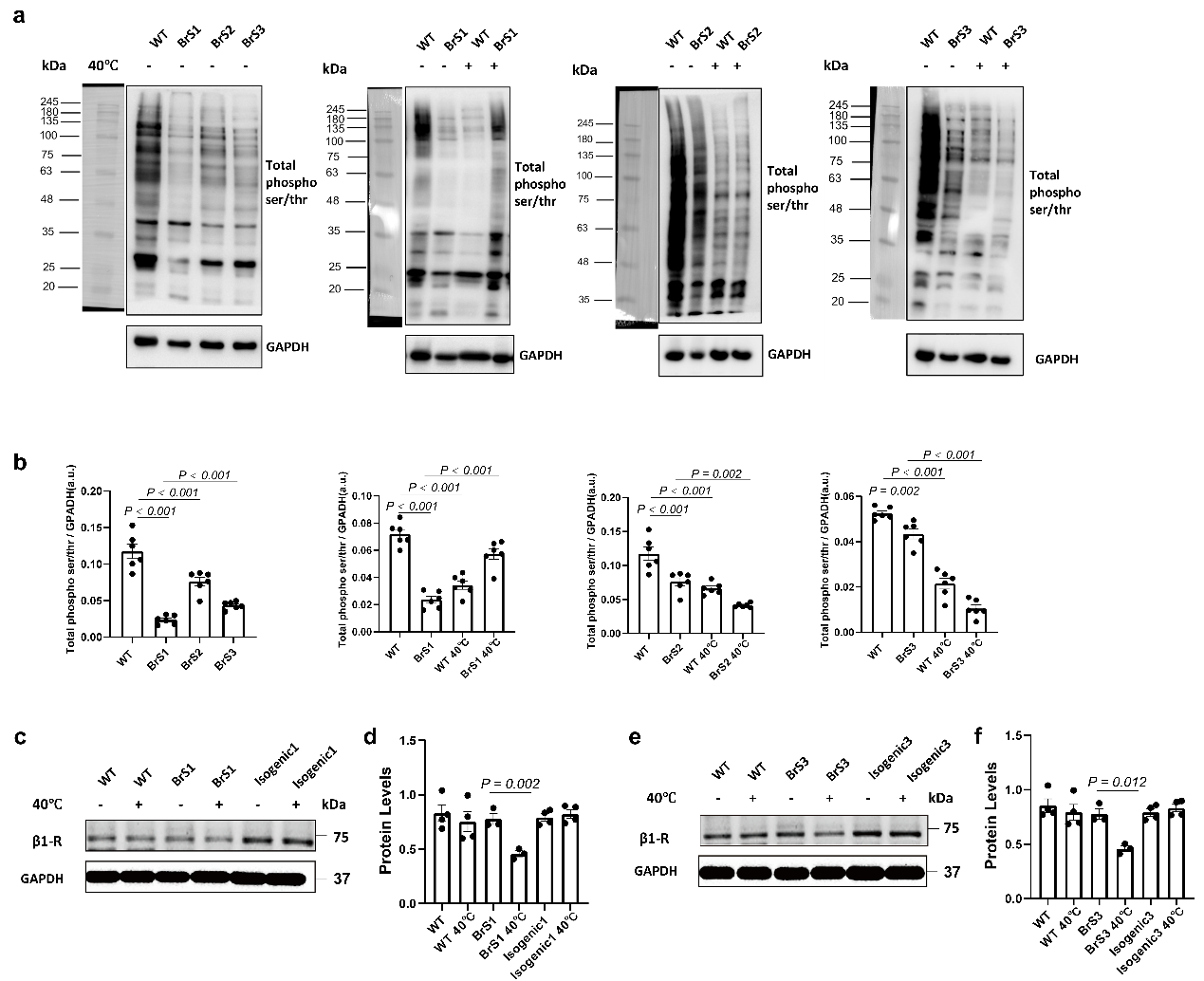


Fig. S17.

**Changes of total phosphorylation levels and β1-Adrenergic receptor expression in BrS cell lines with or without hyperthermia treatment.** Total phosphorylation was measured in hiPSC-CMs from a healthy donor (WT), a BrS-patient with the variant in *SCN10A* (BrS1), a BrS-patient with the variant in *CACNB2* (BrS2) and a BrS-patient with the variant in *SCN5A* (BrS3) at 37℃ and 40℃ for 24 h. (a) and (b) Representative (a) and statistical data (b) of western blot for measuring total phosphorylation in WT and three BrS cell lines at 37℃ (n=3, number of independent experiments) or 40℃ (n=6, number of independent experiments). (c) and (d) Representative (c) and statistical data (d) of western blot for measuring β1-Adrenergic receptor expression in WT and BrS1 cell lines, n=4 (number of independent experiments). (e) and (f) Representative (e) and statistical data (f) of western blot for measuring β1-Adrenergic receptor expression in WT and BrS3 cell lines, n=4 (number of independent experiments). Full-length blots are presented in Supplementary Western blots. The p values are determined by One way-ANOVA with Holm-Sidak post-test.


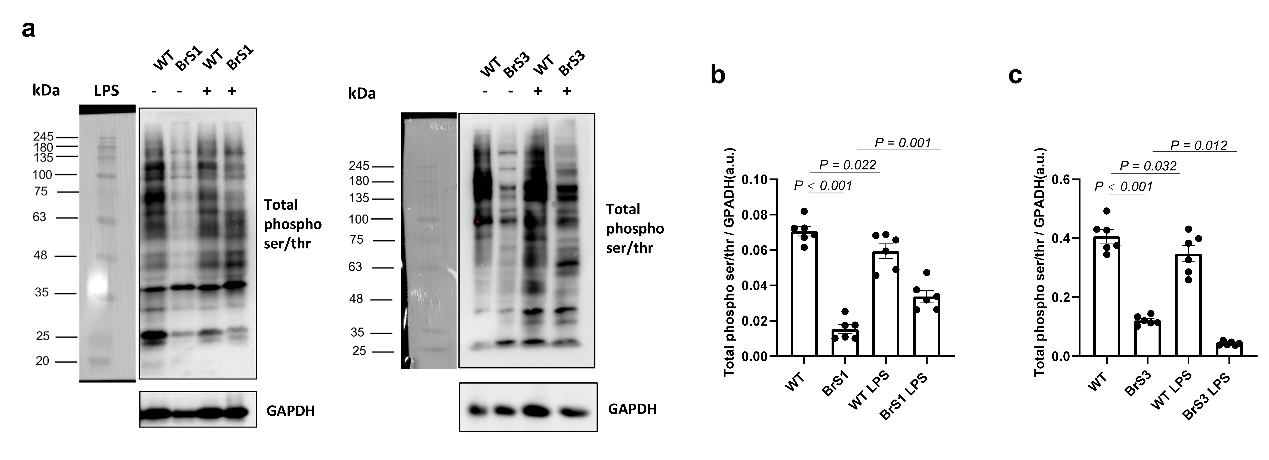


Fig. S18.

**Changes of total phosphorylation levels in BrS cell lines with or without LPS treatment.** Total phosphorylation was measured in hiPSC-CMs from a healthy donor (WT), a BrS-patient with the variant in *SCN10A* (BrS1) and a BrS-patient with the variant in *SCN5A* (BrS3) in presence of LPS (2 μg/ml, 24 hours). (a) Representative and (b-c) statistical data of western blot for measuring total phosphorylation in BrS1 and BrS3 cell lines in presence of LPS, n=6 (number of independent experiments). Full-length blots are presented in Supplementary Western blots. The p values are determined by One way-ANOVA with Holm-Sidak post-test.

**The different effects of hyperthermia challenge on electrophysiological characteristics among 3 BrS cell lines**

|  | BrS1 | BrS2 | BrS3 |
| --- | --- | --- | --- |
| Decreased peak I_Na_ | +* | - | + |
| Abnormal I_Na_ channel gating kinetics |  |  |  |
| Decreased V_0.5_ of activation | + | - | +* |
| Decreased V_0.5_ of inactivation | + | - | + |
| Increased Tau | + | - | - |
| Abnormal action potential |  |  |  |
| Decreased APA | + | - | - |
| Decreased V_max_ | + | - | + |
| Abnormal APD50 | - | + | - |
| Abnormal APD90 | - | - | - |
| Abnormal RP | - | - | - |
| Abnormal calcium transients | + | - | + |
| Potential molecular mechanism | PKA pathway | / | PKA pathway |

**Table S1.**

**The different effects of hyperthermia challenge on electrophysiological characteristics among 3 BrS cell lines.** BrS indicates Brugada syndrome; I_Na_, peak sodium current; V_0.5_, the voltage at which the conductance was half-maximal; Tau, the time constant of channel recovery; APA, action potential amplitude; V_max_, the maximal velocity of depolarization; APD50, action potential duration at 50% repolarization; APD90, action potential duration at 90% repolarization; RP, resting potential; and PKA, the protein kinase A. + means the value in BrS cell line is changed compared to wild type cell line. – means the value in BrS cell line is not changed compared to wild type cell line. * means that the value in BrS cell line is more changed than another BrS cell line with “+”.

**The different effects of LPS challenge on electrophysiological characteristics among 3 BrS cell lines**

|  | BrS1 | BrS2 | BrS3 |
| --- | --- | --- | --- |
| Decreased peak I_Na_ | +* | - | + |
| Abnormal I_Na_ channel gating kinetics |  |  |  |
| Decreased V_0.5_ of activation | + | - | - |
| Increased V_0.5_ of inactivation | + | + | - |
| Increased Tau | + | - | - |
| Abnormal action potential |  |  |  |
| Decreased APA | - | - | - |
| Decreased V_max_ | + | - | +* |
| Abnormal APD50 | - | + | - |
| Abnormal APD90 | - | - | - |
| Abnormal RP | - | - | - |
| Abnormal calcium transients | + | + | + |
| Potential molecular mechanism | ROS/IL-6 |  | ROS/ IL-6 |

**Table S2.**

**The different effects of LPS challenge on electrophysiological characteristics among 3 BrS cell lines.** BrS indicates Brugada syndrome; LPS, lipopolysaccharide; I_Na_, peak sodium current; V_0.5_, the voltage at which the conductance was half-maximal; Tau, the time constant of channel recovery; APA, action potential amplitude; V_max_, the maximal velocity of depolarization; APD50, action potential duration at 50% repolarization; APD90, action potential duration at 90% repolarization; RP, resting potential; ROS, reactive oxygen species; and IL-6, Interleukin-6. + means the value in BrS cell line is changed compared to wild type cell line. – means the value in BrS cell line is not changed compared to wild type cell line. * means that the value in BrS cell line is more changed than another BrS cell line with “+”.
